# Optimization of CEST MRI Reporter Protein Design Using Cation‐Pi Networks

**DOI:** 10.1002/chem.202501638

**Published:** 2025-09-10

**Authors:** David E. Korenchan, Ethan J. French, Emerenziana Runco, Chetan B. Dhakan, Jinwu Yan, Hiroshi Nakashima, Michael T. McMahon, Assaf A. Gilad, Christian T. Farrar

**Affiliations:** ^1^ Athinoula A. Martinos Center for Biomedical Imaging Massachusetts General Hospital Charlestown MA 02129 USA; ^2^ Department of Neurosurgery Harvey Cushing Neuro‐oncology Laboratories, Brigham and Women's Hospital Boston MA 02115 USA; ^3^ F.M. Kirby Research Center for Functional Brain Imaging Kennedy Krieger Institute Baltimore MD 21205 USA; ^4^ Department of Radiology Johns Hopkins School of Medicine Baltimore MD 21287 USA; ^5^ Department of Chemical Engineering and Materials Science Michigan State University East Lansing MI 48824 USA; ^6^ Department of Radiology Michigan State University East Lansing MI 48824 USA

**Keywords:** cation‐pi interaction, chemical exchange saturation transfer, magnetic resonance imaging, nuclear magnetic resonance spectroscopy, reporter proteins

## Abstract

Nucleic acid‐based therapeutics, such as oncolytic virotherapy or gene therapy, would benefit greatly from a reporter gene that induces endogenous production of a protein biomarker to noninvasively track the delivery, persistence, and spread with imaging. Several chemical exchange saturation transfer (CEST) reporter proteins detectable by magnetic resonance imaging (MRI) have been demonstrated to have high sensitivity. However, to date none can provide strong CEST contrast at a distinct resonance from that of endogenous proteins, limiting their specificity. We investigated proteins and peptides containing tyrosine (Tyr), tryptophan (Trp), and lysine (Lys) residues that demonstrate CEST contrast shifted far downfield (4–10 ppm) from water. Although Tyr, Trp, and Lys exchangeable protons are typically not detectable under physiological conditions, those in our tested molecules are, having exchange rates of 400–2500 s^−1^. The large chemical shift dispersion and rapid exchange rates are attributed to unique hydrogen bonding and cation‐π network interactions. These discoveries set the stage for designing a stable reporter protein with high detection specificity and sensitivity that can facilitate the in vivo monitoring of viral and gene therapies using MRI.

## Graphical abstract

A novel engineering approach can produce reporter proteins for cell and viral therapy tracking with unique magnetic resonance imaging (MRI) signatures, detectable with chemical exchange saturation transfer (CEST). We discover how cation‐π interactions between amino acid groups can help us fine‐tune magnetic resonance properties for noninvasive, sensitive, and specific detection of protein reporters.

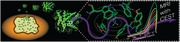

## 1 Introduction

Cell‐ and viral‐based therapeutics hold great promise for targeted treatment of disease. Delivering genetic material to cells using gene transfer, genome editing,^[^ ^1^, ^2^, ^3^, ^4^ ^]^ or oncolytic virotherapy (OV)^[^ ^5^, ^6^, ^7^, ^8^, ^9^ ^]^ has demonstrated great potential, with now 7 adeno‐associated virus (AAV) products receiving FDA approval as of Nov 2023, for Duchenne muscular dystrophy,^[^ ^10^ ^]^ hemophilia A,^[^ ^11^ ^]^ and spinal muscular atrophy,^[^ ^12^ ^]^ as well as a number of other therapeutics in clinical trials.^[^ ^13^, ^14^, ^15^, ^16^, ^17^ ^]^ However, optimizing such biological therapies and assessing their efficacy requires monitoring of the delivery, spread, and persistence of the therapeutic agent. To this end, several reporter gene approaches have been implemented, running the gamut of imaging modalities including optical detection, computed tomography (CT), positron emission tomography (PET), single‐photon emission computed tomography (SPECT), photoacoustic imaging, and magnetic resonance imaging (MRI).^[^ ^18^, ^19^, ^20^, ^21^, ^22^, ^23^, ^24^, ^25^, ^26^, ^27^, ^28^ ^]^ MRI, with its superior anatomical resolution and diversity of contrast mechanisms, is an ideal tool for tracking cellular and viral therapies. Several reviews focusing on MRI reporter gene detection have been published.^[^ ^29^, ^30^, ^31^, ^32^ ^]^

Most imaging approaches rely on the administration of an exogenous substrate or secondary compound that is not accessible to all tissues and requires successive injections for longitudinal imaging. On the other hand, reporter gene proteins containing protons that undergo rapid exchange with the water proton pool are detectable with chemical exchange saturation transfer (CEST) MRI and could eliminate the need for extra injected agents. CEST MRI^[^ ^33^ ^]^ uses frequency‐selective radio‐frequency (RF) pulses to saturate the magnetization of exchangeable protons on molecules such as proteins and metabolites. Proton exchange with bulk water results in a decreased water MRI signal. The CEST contrast depends on the proton exchange rate from solute to water (*k*_sw_), which depends on the exchanging chemical constituent (amide, amine, hydroxyl, etc.) and the surrounding pH, and the volume fraction of the exchangeable proton pool (*f*_s_), which is proportional to the target protein or metabolite concentration. The resonant frequency of the exchangeable protons relative to that of water, called the chemical shift dispersion, also affects the CEST contrast, and along with the chemical exchange rate it provides selectivity for distinguishing the molecule of interest from other metabolite and protein signals. CEST is attractive for monitoring viral therapies since it does not rely on the interaction of the reporter protein with exogenous substrates, radiotracers, or contrast agents.

Several previous studies have used amide proton‐based reporter genes engineered into cells or viruses with fine‐tuned amide exchange properties to successfully detect viral infection or gene expression. These include the lysine‐rich protein (LRP) reporter, which provides amide proton‐based CEST contrast at 3.5 ppm downfield from the water resonance,^[^ ^34^ ^]^ a CEST polypeptide sensitive to Protein Kinase A phosphorylation,^[^ ^35^ ^]^ a modified human protamine‐1 variant with an enhanced CEST signature,^[^ ^36^ ^]^ and supercharged green fluorescent protein (GFP) analogs, enriched with lysine and arginine residues, which have guanidinium proton‐based CEST contrast at 1.8 ppm downfield of water.^[^ ^37^ ^]^ A “multi‐color” polypeptide platform for reporter protein design was also demonstrated.^[^ ^38^ ^]^ The LRP reporter was specifically designed to have much faster amide proton chemical exchange (400 s^−1^)^[^ ^39^ ^]^ than endogenous proteins (30 s^−1^).^[^ ^40^ ^]^ In vivo detection of LRP using CEST imaging has been used to monitor oncolytic viral infection of rat glioma cells in an orthotopic syngeneic animal model,^[^ ^41^ ^]^ detect tumor‐specific LRP expression in genetically modified implanted rat 9 L glioma cells,^[^ ^42^ ^]^ and assess gene therapy in mouse myocardium using an AAV9 viral vector.^[^ ^43^ ^]^ Enhanced amide CEST contrast has been derived from a redesigned LRP reporter protein with improved DNA stability and protein expression.^[^ ^44^ ^]^ More recently, machine learning‐driven protein evolution using short‐peptide CEST measurements has been used to optimize the chemical exchange rate, and hence sensitivity, of amide proton‐based reporters.^[^ ^45^, ^46^, ^47^ ^]^

However, the advantage of amide‐based reporter protein CEST also becomes its Achilles’ heel, for although the proteins have high CEST contrast, there is also a high in vivo background, which must be disentangled from the desired reporter protein signals. In addition, these background CEST signals can change with disease progression or in response to therapy when pH or protein synthesis is altered,^[^ ^48^ ^]^ complicating CEST signal interpretation. A reporter protein with CEST contrast in spectral regions where no endogenous CEST contrast is observed would, therefore, be a great boon for the selective detection of a reporter protein. In this study, we investigated CEST signals from ring‐deshielded exchangeable protons involving tyrosine (Tyr, Y) and tryptophan (Trp, W),^[^ ^33^, ^49^, ^50^, ^51^, ^52^, ^53^, ^54^, ^55^, ^56^, ^57^, ^58^, ^59^ ^]^ shifted far downfield of water and endogenous amide CEST resonances, via ^1^H nuclear magnetic resonance (NMR) spectroscopy for a variety of peptide sequence motifs and proteins. We discovered that cation‐π binding between lysine (Lys, K) and aromatic residues generates rapidly exchanging protons (>1100 s^−1^), leading to higher CEST contrast. Such cation‐π structures could be engineered into a reporter protein that is easy to detect and distinguish from endogenous CEST contrast, breaking the sensitivity and specificity barrier of CEST‐based reporter proteins.

## 2 Results and Discussion

### 2.1 Tyrosine and Tryptophan Proton Exchange Characterization

**Figure 1:**
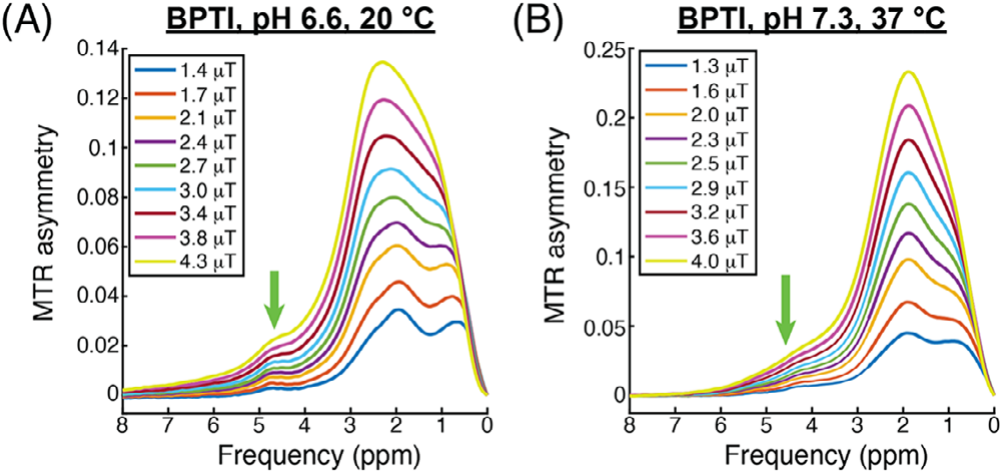
Tyr phenol OH exchange in the protein BPTI using ^1^H CEST NMR spectroscopy. (A‐B) *MTR*_asym_ plots as a function of saturation pulse amplitude (in µT) of BPTI dissolved in phosphate‐buffered saline at A) pH 6.6, 20 °C; and B) pH 7.3, 37 °C. The green arrow indicates the location of the Tyr phenol OH proton exchange contribution.

**Figure 2:**
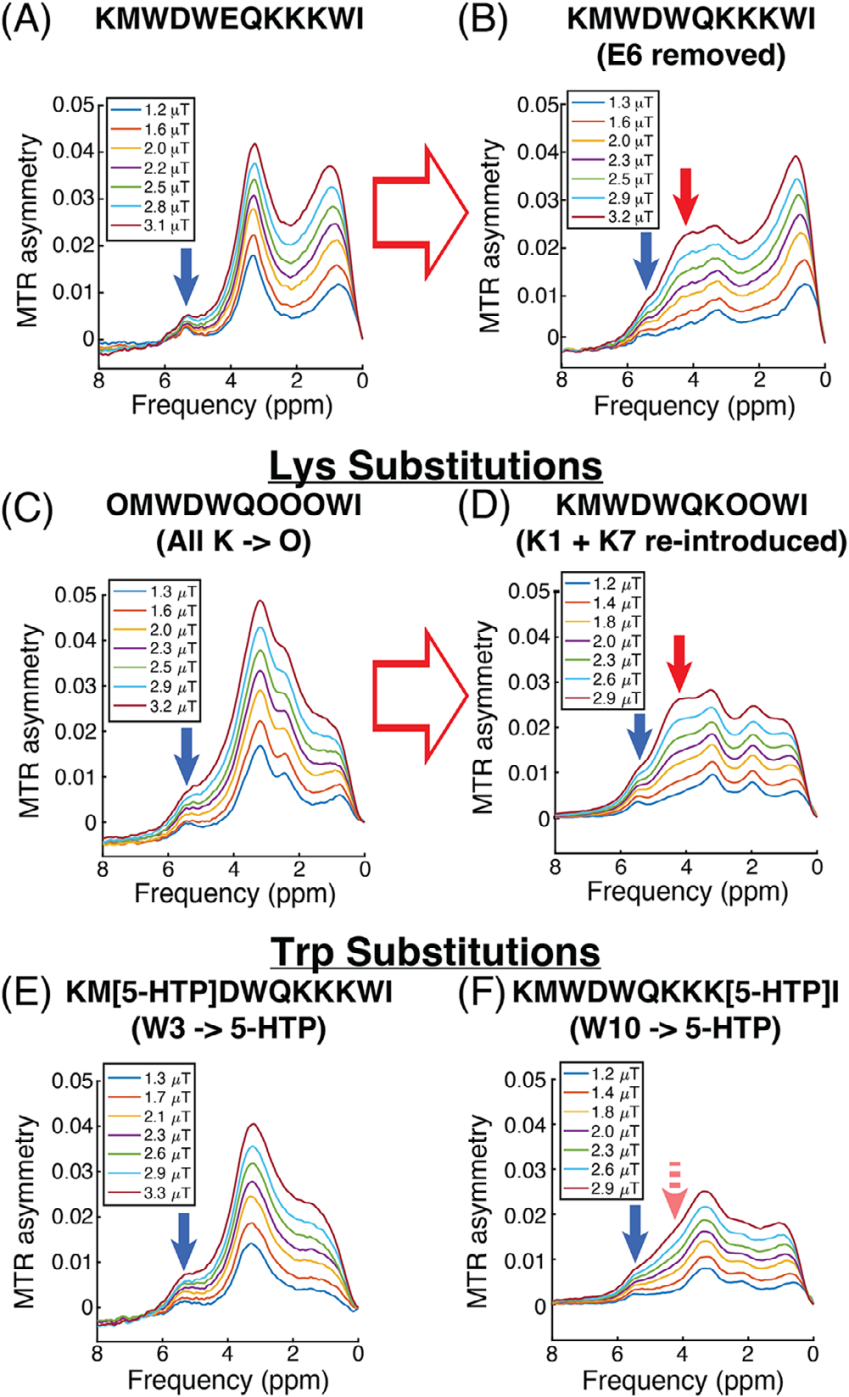
Discovery of a new CEST proton pool at 4.2 ppm in short Trp‐containing peptides. (A‐B) *MTR*_asym_ profile versus saturation pulse amplitude for two peptides, one with A) an E6 residue; and B) the same but with E6 removed. The red and blue arrows indicate the 4.2 ppm and Trp indole NH proton pools, respectively. (C‐F) Investigation into the source of the 4.2 ppm CEST signal origin: *MTR*_asym_ profiles of peptides with amino acid substitutions. C) All K residues substituted for Orn (O). D) Re‐introduction of K at positions 1 and 7. (E‐F) Substitution of E) the third Trp residue; or F) the tenth Trp residue for 5‐hydroxytryptophan (5‐HTP). All peptides were at a concentration of 6.67–8.17 mg/mL.

Exchangeable protons on aromatic rings, such as Tyr and Trp, have been shown to have large chemical shift dispersion. We therefore examined whether proteins and peptides containing such aromatic amino acid residues can generate detectable CEST contrast, starting with the Tyr phenol protons in the bovine pancreatic trypsin inhibitor (BPTI) protein. We studied how the CEST spectrum changed at pH values of 6.6, 7.0, and 7.3, as well as temperatures of 10, 20, and 37 °C (Figure S1). Two representative combinations of sample conditions are presented in Figure 1. A distinct CEST peak is observed in the *MTR*_asym_ profile at ∼4.8 ppm, in good agreement with previous reports.^[^ ^54^, ^55^, ^56^ ^]^ While the peak is easily distinguishable at pH 6.6 and 20 °C (Figure 1A), at physiological pH and temperature (pH 7.3, 37 °C) the signal broadens out and is difficult to resolve from the amide peak (Figure 1B). We quantified the Tyr phenol OH proton exchange rate, *k*_sw_, using QUantification of Exchange rate using varying Saturation Power (QUESP) analysis. In general, the observed Tyr CEST peak broadened out as the temperature increased (Figure S1). Faster exchange can shuttle the saturation effect to the water peak more rapidly and increase CEST sensitivity; however, too large of an exchange rate relative to the chemical shift dispersion from water can decrease the saturation efficiency on the proton in question, undoing the sensitivity gains.^[^ ^60^ ^]^ This is likely the case with endogenous Tyr phenol OH exchange. A previous NMR study of Tyr phenol OH proton exchange in BPTI at 4 °C (see Figures 2 and 4 of reference^[^ ^55^ ^]^) reported that exchangeable phenol protons from four Tyr residues (Y10, Y21, Y23, and Y35) can be observed in ^1^H NMR spectra, and their relative contributions change as both the temperature and pH are varied.^[^ ^55^ ^]^ The observation of a very broad CEST peak at pH 7.3 and 37 °C (Figure 1B) suggests that Tyr cannot provide sufficient in vivo contrast unless the proton exchange can be slowed down. We are currently working on designing BPTI variants to modulate the exchange rate of the Tyr phenol OH protons via side‐chain interactions.

Because Trp also contains exchangeable protons downfield of typical in vivo CEST resonances, we then measured the CEST contrast from Trp exchangeable indole NH protons in a series of peptides, all derived from a base peptide sequence KMWDWEQKKKWI via amino acid deletions, substitutions, and rearrangements. All the peptides exhibited a distinct peak from the Trp indole NH proton at ∼5.4 ppm (Figure S2A), as well as a strong peak at ∼3.5 ppm corresponding with the amide backbone NH protons.^[^ ^40^ ^]^ The mean ± s.d. indole NH proton exchange rate across all peptides was 736 ± 189 s^−1^ and covered a range from 400–1200 s^−1^ (Table S2). These values are significantly larger than those reported in the literature^[^ ^49^, ^50^, ^51^, ^53^ ^]^ (on the order of 10 s^−1^), presumably due to the higher temperature and/or different solution conditions (e.g., phosphate buffer) used in our study. Indeed, inorganic phosphate strongly catalyzes amine and hydroxyl proton exchange in amino acids.^[^ ^56^ ^]^ The observation that the Trp indole NH CEST peak linewidth generally remained the same at the lowest saturation amplitudes, ω_1_ (below 600 rad s^−1^; see Figure S2A and Figure 2A) suggests that *k*_sw_ > ω_1_ in this range^[^ ^60^, ^61^ ^]^ and, therefore, corroborates our unexpectedly high Trp indole NH *k*_sw_ measurements.

### 2.2 Investigation Into Cation‐π Effects on Peptide CEST

One of the Trp‐containing peptides showed an additional CEST resonance not observed in any of the other peptide sequences. Figure 2 shows the characterization of this new exchangeable proton pool. When the glutamate (Glu, E) was removed from the base peptide sequence (KMWDWEKKKWI; CEST profile shown in Figure 2A), an additional CEST pool appeared at ∼4.2 ppm, between the Trp indole NH and amide/amine proton resonances (Figure 2B). The CEST signal from this pool was comparable in magnitude to the nearby amide proton pool (3.5 ppm). QUESP fitting of the peaks in the CEST spectrum revealed that the protons giving rise to the 4.2 ppm resonance exchanged with a rate constant *k*_sw_ = 1930 s^−1^, significantly higher than the exchange rate of the Trp indole NH or amide protons in the same peptide (Figure S3B‐C).

We hypothesized that the new CEST signal arose from the interaction between Trp and one of the Lys residues in the peptide, since Lys amine proton exchange could be rapid enough to explain the large exchange rate. To confirm this hypothesis, we tested various modifications of the peptide giving the 4.2 ppm signal. Replacing all Lys (K) residues in the peptide with ornithine (Orn, O), which has a nearly identical amine‐containing side‐chain but with one fewer carbon, abolished the 4.2 ppm peak (Figure 2C). The 4.2 ppm signal then returned when Lys was re‐substituted for residues O1 and O7 (Figure 2D). Replacing only K7 while leaving O in all other positions did not bring the 4.2 ppm peak back (Figure S4A). These results suggested that the origin of the 4.2 ppm CEST signal was the K1 side‐chain amine proton. The 4.2 ppm peak intensity was also affected when either the W3 or W10 residue was replaced with 5‐hydroxytryptophan (5‐HTP) (Figure 2E‐F), in further support of our hypothesis. While W3 substitution with 5‐HTP abolished the 4.2 ppm peak (Figure 2E), W10 substitution significantly reduced it, but did not eliminate it completely (Figure 2F). The dependence of the 4.2 ppm peak on the W3 and W10 residues suggests that they form cation‐π interactions with the K1 side‐chain amine, a type of interaction that occurs often in biological proteins.^[^ ^62^ ^]^ The added hydroxyl moiety in 5‐HTP is a small change in the peptide, but it likely disrupts the cation‐π interaction by direct hydrogen bonding with the K1 amine. The NMR results further implied that the K1 interacts more strongly with W3 than with W10 (see Figure 2E‐F). The exchange rate of Lys side‐chain amine protons is typically too fast to detect by CEST under physiological conditions;^[^ ^39^, ^56^ ^]^ therefore, the cation‐π interaction between the K1, W3, and W10 residues in the 11‐mer peptide must both induce an increased downfield chemical shift of the amine proton and reduce the exchange rate.

**Figure 3:**
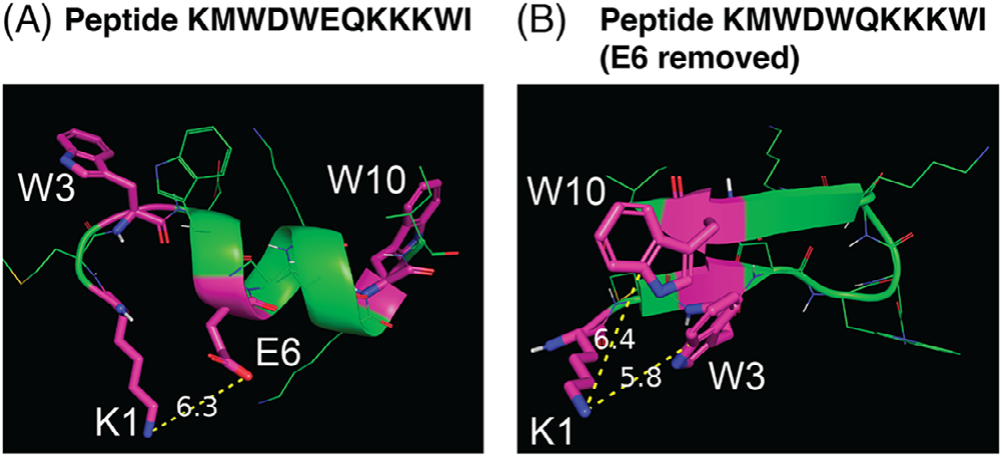
Predicted peptide structures for the peptide CEST data displayed in Figure 2A‐B. A) Peptide sequence KMWDWEQKKKWI. B) Peptide sequence KMWDWQKKKWI (same, but with the E6 residue removed). Side‐chains of interest are drawn with thicker lines, highlighted in magenta, and labeled with the single‐letter amino acid and numerical position. Distances between atoms are in units of Angstroms.

Next, we performed peptide structure prediction to confirm our hypothesis that a cation‐π interaction gave rise to the 4.2 ppm CEST signal. Figure 3 displays the lowest‐energy conformers for the two peptides corresponding with the CEST profiles in Figure 2A‐B. When the peptide contained a Glu residue (E6), the K1 side‐chain amine localized near the E6 carboxyl, and the W3 and W10 side‐chains were located far from the two (Figure 3A). The predicted conformer with the second‐lowest energy shared this property as well (Figure S5A). When the E6 residue was removed, however, the K1 and W3 side‐chains ended up close to one another. Additionally, the peptide backbone looped around and the W3 and W10 side‐chains came quite close together (Figure 3B). The significant changes in peptide structure and in the Trp side‐chain interactions upon E6 removal are corroborated by both CD and fluorescence spectroscopy, which are greatly altered between the two peptides (Figure S6 and S7). Considering the differential effects of substituting 5‐HTP for Trp on the CEST NMR profile (Figure 2E‐F), we suggest that the W10 side‐chain stabilizes the K1‐W3 interaction, perhaps with the K1 amine ending up sandwiched between the W3 and W10 indole rings, similar to what has been observed in some proteins.^[^ ^62^ ^]^ Although none of the other four predicted structures had the W3 and W10 side‐chains in close proximity to one another, the highest‐energy predicted conformer did involve a short distance between the K1 and W3 side‐chains (Figure S5B). This observation also agrees with the stronger effects of the W3 versus W10 residue on the 4.2 ppm peak intensity. The results suggest that cation‐π interactions may be favored in the E6‐removed peptide, producing the unique CEST signature at 4.2 ppm seen in Figure 2B.

### 2.3 Evaluation of Glucoamylase as a CEST‐Detectable Reporter Protein Platform

**Figure 4:**
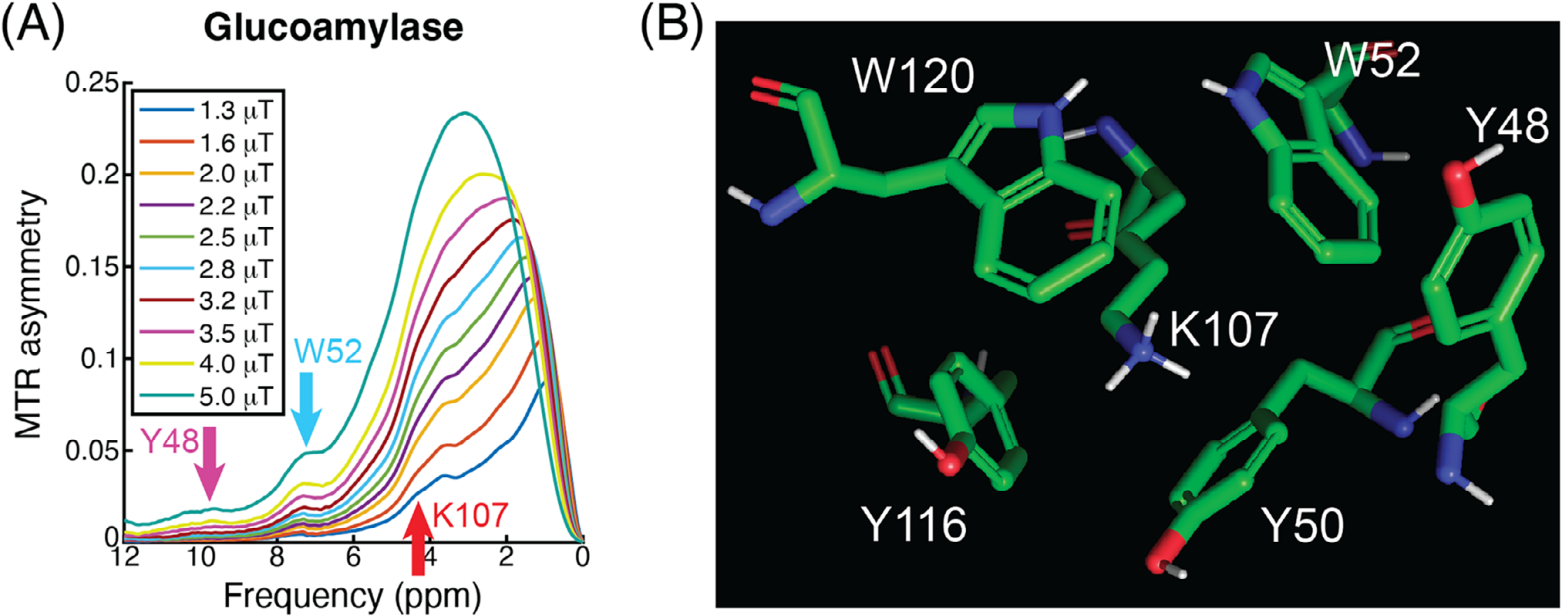
A) ^1^H CEST NMR contrast obtained from a solution of glucoamylase protein (57.5 mg/mL) at pH 7.3 and 37 °C. The magenta and light blue arrows indicate the signals at 9.8 ppm and 7.3 ppm, respectively. Tentative peak assignments are shown next to each arrow. B) Amino acid side‐chains of glucoamylase displaying cation‐π interactions and likely giving rise to the CEST pools observed in (A).

A previous study identified a number of proteins in the protein data bank that contained cation‐π networks.^[^ ^62^ ^]^ One of the most striking examples is the protein glucoamylase, a fungal protein from the *Rhizopus* and *Aspergillus* genera.^[^ ^62^ ^]^ The protein contains 475–512 amino acids in its cleaved state including 15 Trp residues and 21 Tyr residues.^[^ ^63^ ^]^ Within the protein is a Lys side‐chain sandwiched between two Tyr and two Trp ring structures (Figure 4B). A previous ^1^H NMR study of glucoamylase showed several highly downfield‐shifted peaks, appearing 4–10 ppm downfield of water.^[^ ^63^ ^]^ Several of these peaks were attributed to amino acids within or near the Lys‐Tyr_2_‐Trp_2_ complex and were shown to vary in intensity as the pH of the solution was varied. The peaks also disappeared from the ^1^H NMR spectrum entirely when the protein was dissolved in D_2_O rather than H_2_O, confirming that they arise from exchangeable protons. We expected that the peaks shifted downfield by the cation‐π interactions would lead to significant, downfield‐shifted CEST signatures.

Figure 4 shows CEST profiles for the proton pools present in a solution of glucoamylase under physiological conditions. CEST resonances are observed at 4.2, 7.3, and 9.8 ppm downfield of the water resonance. The 4.2 ppm signal is quite broad and poorly resolved from the amide proton signal at 3.5 ppm, although it can be distinguished at lower saturation amplitudes (Figure 4A). We believe this signal arises from a Lys‐Trp interaction like that observed in the Trp‐containing short peptides (Figure 2B), perhaps involving K107 (see Figure 4B). The 7.3 ppm peak is the most pronounced, giving a larger and narrower signal than at 9.8 ppm (Figure 4A). We measured the exchange rate of the 7.3 ppm peak to be 2470 s^−1^ (Figure S8D). The previous NMR study of glucoamylase and various mutants observed that the 7.3 ppm peak disappeared for the W52F mutant protein,^[^ ^63^ ^]^ suggesting that the 7.3 ppm peak is due to W52. The exchange rate of the broader 9.8 ppm peak was challenging to quantify since the very broad peak suggests a very fast exchange rate, significantly higher than the maximum saturation amplitude used (ω_1_ = 1500 rad s^−1^). Nuclear Overhauser enhancement (NOE) cross‐peaks observed in the NMR study^[^ ^63^ ^]^ led the authors to suggest that the 9.8 ppm peak is due to a Tyr residue, possibly Y48, which is located close to the Lys‐Tyr_2_‐Trp_2_ π‐bonding complex. When the sample temperature was lowered from 37 °C to 20 °C, the downfield Tyr peak at 9.8 ppm appeared to narrow, likely due to a reduction in the extremely fast chemical exchange rate of the phenol OH, whereas the upfield 7.3 ppm resonance was better resolved at 37 °C, due to an increased exchange rate of the much slower exchanging indole NH (Figure S8A‐B). We note that no distinct endogenous CEST peaks have been reported in the literature at chemical shifts greater than 4 ppm in cell lysates,^[^ ^64^ ^]^ intact cells,^[^ ^65^ ^]^ or in vivo tissue.^[^ ^66^ ^]^ Consistent with these earlier studies, we have observed no significant CEST peak beyond 4 ppm during in vivo CEST spectroscopic imaging in a mouse brain (Figure S11B). We thus confirmed that glucoamylase gives strong CEST contrast that can be easily discriminated from the endogenous protein background signal under near‐physiological conditions.

**Figure 5:**
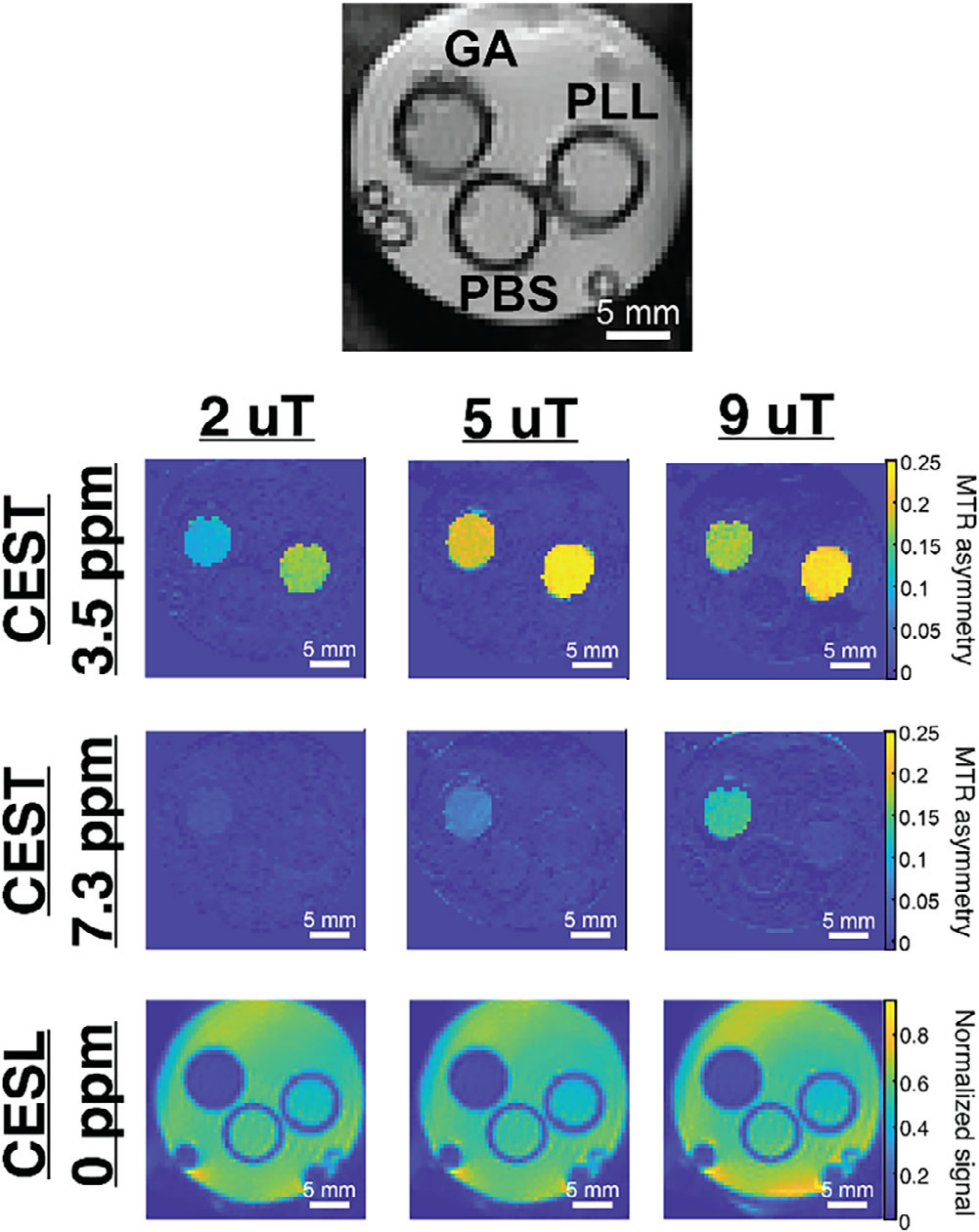
CEST and water‐resonant CESL imaging comparison of glucoamylase and poly‐L‐lysine contrast at different saturation/locking amplitudes. CEST images are shown at the amide (3.5 ppm, top row) and cation‐π shifted (7.3 ppm, middle row) proton chemical shifts. CESL images with 400 ms of water spin‐locking are shown in the bottom row. Both proteins were at 597 µM and pH 7.3. The unsaturated image is shown at the top.

Although the highly‐shifted CEST contrast from the glucoamylase protein requires further improvement by protein design before in vivo implementation, we wanted to predict how well we might be able to observe the signal in vivo. We therefore performed a CEST imaging experiment to compare glucoamylase with poly‐L‐lysine (PLL), a standard amide CEST agent. A similar lysine‐rich protein has been used in prior in vivo studies.^[^ ^34^, ^41^ ^]^ Figure 5 displays the results. While the amide (3.5 ppm) contrast for both proteins peaks at a 5 µT saturation amplitude, the highly‐shifted glucoamylase contrast continues to increase with larger saturation amplitudes. Although at 2 µT the PLL amide contrast is substantially higher than that of the glucoamylase Trp indole NH, it should be noted that PLL has an average of 64 amide protons per molecule, whereas we expect that the glucoamylase has only one indole NH proton from the W52 residue. This means that the protons at 7.3 ppm give 5.3‐fold more contrast per proton than do the PLL protons at 3.5 ppm. This factor increases to 17‐fold and 36.6‐fold at 5 µT and 9 µT saturation, respectively (see Table S3).

While the fast exchange rate of the Trp indole NH does provide dramatically increased sensitivity, the large saturation powers required to efficiently saturate such rapidly exchanging protons will complicate clinical translation, where saturation power is limited by constraints on RF tissue absorption and RF amplifier power. We therefore anticipate that further fine‐tuning of the cation‐π structure to reduce the exchange rate will be required to optimize the detection sensitivity for the clinical scenario. Alternatively, chemical exchange spin‐locking (CESL) on the water resonance has been demonstrated to have significantly higher sensitivity than CEST for detecting exchangeable protons in the intermediate to fast exchange regime (*k*_ex_ ≥ Δω) and does not require high spin‐lock powers.^[^ ^60^ ^]^ This is demonstrated in Figure 5 where dramatically higher contrast‐to‐noise ratio (CNR) is observed for glucoamylase with CESL (400 ms locking time) than with CEST. The CESL contrast varied little with the spin‐lock amplitude, as predicted by theory.^[^ ^60^ ^]^ Compared with the tube containing PBS, the glucoamylase signal was 89.7% lower across all locking field amplitudes, compared with only 14.6% lower for the PLL. While all exchangeable protons contribute to the CESL contrast, those with a large chemical shift dispersion from water and a faster exchange rate dominate the CESL contrast.^[^ ^60^ ^]^ This can be seen by comparison with the very low CESL CNR observed for PLL, where the slow amide proton exchange rate (*k*_ex _= 579 s^−1^, determined from our experimental data with the inverse QUESP method) and small chemical shift (Δω = 3.5 ppm) result in very little CESL contrast. We expect, therefore, that the CESL contrast for glucoamylase is mainly dictated by the protons at 7.3 and 9.8 ppm. We also note that the relative independence of spin‐lock contrast on the spin‐lock pulse amplitude makes CESL ideal for clinical translation, where large RF amplitudes are prohibitive and RF field inhomogeneity may be large.

Similarly large downfield CEST shifts to those we report here have been seen for the aspartate‐histidine‐serine (Asp‐His‐Ser) catalytic triad of bovine chymotrypsinogen‐A.^[^ ^67^ ^]^ Two proton exchange peaks were observed with maximal shifts of about 8 and 13 ppm downfield of water, attributed to the Ser195‐Hε2 and His57‐Hδ1 protons, respectively. However, the CEST signal was relatively weak and only observed at pH < 7 and temperatures ≤ 20 °C. A third exchange peak from Ser195‐Hγ was observed above pH 7, resonating at ∼7 ppm downfield of water, but no spectra were presented. In contrast, the glucoamylase CEST signals we observed are robust at physiological temperature and pH and are therefore ideal for CEST reporter proteins.

Some important considerations are worth mentioning. It is possible that alkali cations might outcompete the amino acid cation. We do not think this is a significant concern in most biological contexts for a couple of reasons. First, cation‐π interactions exist in many proteins under physiological conditions, suggesting that metal cations do not interfere with them. Second, although potassium (K^+^) can disrupt cation‐π interactions between amino acid side‐chains^[^ ^68^ ^]^ and its intracellular concentration is ∼155 mM,^[^ ^69^ ^]^ other hydrophilic, negatively‐charged groups nearby should be able to prevent K^+^ disruption. The compartmental pH would likely also play a role in the contrast, both regarding the offset of the peak and the exchange rate, which in turn would affect the labeling efficiency of the saturation pulses. Therefore, if a reporter gene product is designed for detection within a near‐neutral pH compartment such as the cytosol, some design considerations may be necessary to prevent localization to lysosomes. Another major consideration regarding the CEST contrast is the water accessibility of the cation‐π interaction, since a cation‐π network buried within the interior of the reporter protein would give significantly lower contrast. Other literature sources suggest, however, that the majority of cation‐π interactions in biological proteins occur on or near the surface of the protein, despite the hydrophobicity of the aromatic amino acids involved.^[^ ^62^ ^]^ This is encouraging for the design of a stable reporter protein in the future.

Another important consideration regards the optimal saturation parameters to best detect the cation‐π shifted CEST resonances. We expect the optimal saturation duration to be similar to amide CEST because the CEST effect buildup rate depends mainly upon the spin‐lattice relaxation time constant (*T*_1_) of water.^[^ ^60^ ^]^ The very fast exchange rate of the 7.3 ppm pool (2470 s^−1^; Figure S8D) dictates that the saturation pulse amplitude should be as large as possible without exceeding tissue specific absorption rate (SAR) limits to maximize the labeling efficiency and, thus, the contrast.^[^ ^60^ ^]^ We note that, in contrast to amide‐based reporters with small chemical shifts, the large chemical shift of cation‐π based reporters allows for the use of high saturation powers without inducing significant direct water saturation, or spillover effect, which can confound the CEST contrast.^[^ ^60^ ^]^

One important concern regarding reporter proteins is their immunogenicity, since foreign proteins in the body eventually elicit an immune response. The proteins can be presented by antigen‐presenting cells (APCs) with major histocompatibility complex (MHC) class I or II, depending on how they are encountered in the body.^[^ ^70^, ^71^ ^]^ We and others have observed no effects of CEST‐based reporters in vivo, and this may be due to their expression being limited to the cytoplasm, which slows the host immune system's response. It is also possible that oncolytic virotherapy may particularly benefit from the immunogenicity of a reporter protein by stimulating a more rapid anti‐tumor immune response.^[^ ^41^, ^44^ ^]^

## 3 Conclusion

The short peptides and glucoamylase studied in this work demonstrate the great potential of cation‐π networks for generating specific (large chemical shift) and sensitive (fast exchange rate) contrast for reporter protein detection, although they are not suitable for in vivo translation. Future studies will aim to isolate optimized cation‐π structures and engineer multiple repeats within a reporter protein to maximize selective CEST contrast. By investigating proteins likely to provide strong, highly downfield‐shifted CEST contrast and combining multiple protein domains into a stable protein, we will be poised to develop a reporter protein that will be extremely sensitive and specific, enabling clear recognition of in vivo viral activity for therapeutic applications and providing a crucial tool for the development and evaluation of cell‐ and viral‐based therapies.

## 4 Experimental Section

*Chemical reagents and sample preparation*: All synthetic peptides were obtained from GenScript (Piscataway, NJ, USA). All proteins was purchased from Sigma Aldrich (St. Louis, MO, USA). All peptide and protein samples were prepared with 1x phosphate‐buffered saline along with 1 mM of sodium formate (concentration reference). For all samples but the BPTI, the pH was adjusted to 7.30 ± 0.05 at room temperature by titrating with either HCl or NaOH.

*^1^H NMR spectroscopy and CEST measurements*: All samples were measured using a 14 T (600 MHz) Bruker NMR spectrometer. The water *T*_1_ was measured using an inversion‐recovery pulse sequence with the z‐gradient on to minimize radiation damping.^[^ ^72^ ^]^ CEST z‐spectroscopy was performed using an ultrafast z‐spectroscopy sequence.^[^ ^73^ ^]^

*CEST, CESL imaging*: Imaging was performed on a 9.4T Bruker small‐bore scanner. Three 700 µL tubes containing the imaging samples were kept at 37 °C during imaging. Following prescans, coil calibration, and shimming, a single‐shot 2D echo‐planar imaging (EPI) sequence (0.47 × 0.47 × 5 mm^3^ voxel size) was performed at saturation offsets out to ±8 ppm. The imaging was repeated for five saturation amplitudes: [2,3,5,7,9] µT. Spin‐locking on water for CESL contrast was performed with the same EPI sequence and pulse amplitudes, and at twelve locking durations: [50,100,150,200,300,400,500,750,1000,2000,3000,5000] ms.

*NMR data processing*: Ultrafast z‐spectroscopy and imaging CEST data were analyzed using custom scripts written in MATLAB (MathWorks, Natick, MA, USA). The *MTR*_asym_ was calculated as:

(1) $$\;MTR_{asym}=Z\left(-\delta \right)-Z\;\left(\delta \right)=\frac{S\left(-\delta \right)}{S_{ref}\left(-\delta \right)}\;-\frac{S\left(\delta \right)}{S_{ref}\left(\delta \right)}$$

Here, S(δ) and S(−δ) refer to the signal magnitude on opposite sides of the water resonance at a chemical shift of magnitude δ, and $S_{ref}$ is the signal profile obtained without saturation. Individual CEST peaks in the z‐spectrum were also fitted using Pseudo‐Voigt lineshapes.^[^ ^74^ ^]^ Using the fitted lineshape amplitudes, the proton volume fraction (*f*_s_) and exchange rate (*k*_sw_) for each exchangeable proton pool were then determined with QUESP.^[^ ^39^, ^75^ ^]^

*Computational peptide structure prediction and display*: Peptide structure prediction was performed using the PEP‐FOLD4 algorithm.^[^ ^76^, ^77^, ^78^, ^79^ ^]^ The resulting .pdb structure files were downloaded and visualized using PyMOL software (Schrödinger, New York, NY, USA).

## Supporting Information

The authors have cited additional references within the Supporting Information.^[^ ^80^, ^81^, ^82^, ^83^ ^]^

## Conflict of Interest

The authors declare no conflict of interest.

## Supporting information



Supporting Information

## Data Availability

The data that support the findings of this study are available from the corresponding author upon reasonable request.

## References

[1] I. Lostalé‐Seijo , J. Montenegro , Nat. Rev. Chem. 2018, 2, 258.

[2] D. Sharma , S. Arora , J. Singh , B. Layek , Int. J. Biol. Macromol. 2021, 183, 2055. 34087309 10.1016/j.ijbiomac.2021.05.192 PMC8266766

[3] Y. Sung , S. Kim , Biomater. Res. 2019, 23, 8. 30915230 10.1186/s40824-019-0156-z PMC6417261

[4] M. R. Cring , V. C. Sheffield , Gene Ther. 2022, 29, 3. 33037407 10.1038/s41434-020-00197-8

[5] H. L. Kaufman , F. J. Kohlhapp , A. Zloza , Nat. Rev. Drug Discov. 2015, 14, 642. 26323545 10.1038/nrd4663 PMC7097180

[6] N. Macedo , D. M. Miller , R. Haq , H. L. Kaufman , J. Immunother. Cancer 2020, 8, e001486. 33046622 10.1136/jitc-2020-001486 PMC7552841

[7] D. Lin , Y. Shen , T. Liang , Sig. Transduct. Target. Ther. 2023, 8, 156. 10.1038/s41392-023-01407-6 PMC10090134 37041165

[8] S. Z. Shalhout , D. M. Miller , K. S. Emerick , H. L. Kaufman , Nat. Rev. Clin. Oncol. 2023, 20, 160. 36631681 10.1038/s41571-022-00719-w

[9] A. L. Ling , I. H. Solomon , A. M. Landivar , H. Nakashima , J. K. Woods , A. Santos , N. Masud , G. Fell , X. Mo , A. S. Yilmaz , J. Grant , A. Zhang , J. D. Bernstock , E. Torio , H. Ito , J. Liu , N. Shono , M. O. Nowicki , D. Triggs , P. Halloran , R. Piranlioglu , H. Soni , B. Stopa , W. L. Bi , P. Peruzzi , E. Chen , S. W. Malinowski , M. C. Prabhu , Y. Zeng , A. Carlisle , S. J. Rodig , P. Y. Wen , E. Q. Lee , L. Nayak , U. Chukwueke , L. N. Gonzalez Castro , S. D. Dumont , T. Batchelor , K. Kittelberger , E. Tikhonova , N. Miheecheva , D. Tabakov , N. Shin , A. Gorbacheva , A. Shumskiy , F. Frenkel , E. Aguilar‐Cordova , L. K. Aguilar , D. Krisky , J. Wechuck , A. Manzanera , C. Matheny , P. P. Tak , F. Barone , D. Kovarsky , I. Tirosh , M. L. Suvà , K. W. Wucherpfennig , K. Ligon , D. A. Reardon , E. A. Chiocca , Nature 2023, 623, 157. 37853118 10.1038/s41586-023-06623-2 PMC10620094

[10] A. Manini , E. Abati , A. Nuredini , S. Corti , G. P. Comi , Front. Neurol. 2022, 12, 814174. 35095747 10.3389/fneur.2021.814174 PMC8797140

[11] B. J. Samelson‐Jones , L. A. George , Annu. Rev. Med. 2023, 74, 231. 36103998 10.1146/annurev-med-043021-033013 PMC9892335

[12] K. Kotulska , A. Fattal‐Valevski , J. Haberlova , Front. Neurol. 2021, 12, 726468. 34721262 10.3389/fneur.2021.726468 PMC8548432

[13] P. Young , Drug Disc. Today 2023, 28, 103610. 10.1016/j.drudis.2023.103610 37169134

[14] V. V. Kolesnik , R. F. Nurtdinov , E. S. Oloruntimehin , A. V. Karabelsky , A. S. Malogolovkin , Clin. Transl. Med. 2024, 14, e1607. 38488469 10.1002/ctm2.1607 PMC10941601

[15] T. Burdett , S. Nuseibeh , Gene Ther. 2023, 30, 323. 36089633 10.1038/s41434-022-00363-0

[16] H. K. E. Au , M. Isalan , M. Mielcarek , Front. Med. 2022, 8, 809118. 10.3389/fmed.2021.809118 PMC8864161 35223884

[17] D. A. Kuzmin , M. V. Shutova , N. R. Johnston , O. P. Smith , V. V. Fedorin , Y. S. Kukushkin , J. C. M. van der Loo , E. C. Johnstone , Nat. Rev. Drug Discov. 2021, 20, 173. 33495615 10.1038/d41573-021-00017-7

[18] F. Schilling , S. Ros , D.‐E. Hu , P. D'Santos , S. McGuire , R. Mair , A. J. Wright , E. Mannion , R. J. M. Franklin , A. A. Neves , K. M. Brindle , Nat. Biotechnol. 2017, 35, 75. 27918546 10.1038/nbt.3714 PMC5230773

[19] P. Brader , I. Serganova , R. G. Blasberg , J. Nucl. Med. 2013, 54, 167. 23318292 10.2967/jnumed.111.099788

[20] I. Serganova , R. G. Blasberg , J. Nucl. Med. 2019, 60, 1665. 31792128 10.2967/jnumed.118.220004 PMC12079160

[21] S. C. Concilio , S. J. Russell , K.‐W. Peng , Mol. Ther. – Oncolytics 2021, 21, 98. 33981826 10.1016/j.omto.2021.03.006 PMC8065251

[22] T. Gao , P. Wang , T. Gong , Y. Zhou , A. Wang , X. Tang , X. Song , Y. Fan , Int. J. Mol. Sci. 2022, 23, 8443. 35955578 10.3390/ijms23158443 PMC9368793

[23] P. S. Patrick , J. Hammersley , L. Loizou , M. I. Kettunen , T. B. Rodrigues , D.‐E. Hu , S.‐S. Tee , R. Hesketh , S. K. Lyons , D. Soloviev , D. Y. Lewis , S. Aime , S. M. Fulton , K. M. Brindle , Proc. Natl. Acad. Sci. USA 2014, 111, 415. 24347640 10.1073/pnas.1319000111 PMC3890795

[24] R. Weissleder , A. Moore , U. Mahmood , R. Bhorade , H. Benveniste , E. A. Chiocca , J. P. Basilion , Nat. Med. 2000, 6, 351. 10700241 10.1038/73219

[25] A. E. Deans , Y. Z. Wadghiri , L. M. Bernas , X. Yu , B. K. Rutt , D. H. Turnbull , Magn. Reson. Med. 2006, 56, 51. 16724301 10.1002/mrm.20914 PMC4079558

[26] G. Genove , U. DeMarco , H. Xu , W. F. Goins , E. T. Ahrens , Nat. Med. 2005, 11, 450. 15778721 10.1038/nm1208

[27] B. Cohen , H. Dafni , G. Meir , A. Harmelin , M. Neeman , Neoplasia 2005, 7, 109. 15802016 10.1593/neo.04436 PMC1501126

[28] B. Cohen , K. Ziv , V. Plaks , T. Israely , V. Kalchenko , A. Harmelin , L. E. Benjamin , M. Neeman , Nat. Med. 2007, 13, 498. 17351627 10.1038/nm1497

[29] A. A. Gilad , P. T. Winnard Jr , P. C. M. van Zijl , J. W. M. Bulte , NMR Biomed. 2007, 20, 275. 17451181 10.1002/nbm.1134

[30] I. K. Cho , S. Wang , H. Mao , A. W. Chan , Am. J. Nucl. Med. Mol. Imaging 2016, 6, 234. 27766183 PMC5069277

[31] A. Farhadi , F. Sigmund , G. G. Westmeyer , M. G. Shapiro , Nat. Mater. 2021, 20, 585. 33526879 10.1038/s41563-020-00883-3 PMC8606175

[32] K. M. Brindle , Trends Genet. 2022, 38, 996. 35641343 10.1016/j.tig.2022.05.006

[33] K. M. Ward , A. H. Aletras , R. S. Balaban , J. Magn. Reson. 2000, 143, 79. 10698648 10.1006/jmre.1999.1956

[34] A. A. Gilad , M. T. McMahon , P. Walczak , P. T. Winnard , V. Raman , H. W. M. van Laarhoven , C. M. Skoglund , J. W. M. Bulte , P. C. M. van Zijl , Nat. Biotechnol. 2007, 25, 217. 17259977 10.1038/nbt1277

[35] R. D. Airan , A. Bar‐Shir , G. Liu , G. Pelled , M. T. McMahon , P. C. M. van Zijl , J. W. M. Bulte , A. A. Gilad , Magn. Reson. Med. 2012, 68, 1919. 23023588 10.1002/mrm.24483 PMC3504643

[36] A. Bar‐Shir , G. Liu , K. W. Y. Chan , N. Oskolkov , X. Song , N. N. Yadav , P. Walczak , M. T. McMahon , P. C. M. van Zijl , J. W. M. Bulte , A. A. Gilad , ACS Chem. Biol. 2014, 9, 134. 24138139 10.1021/cb400617q PMC3985336

[37] A. Bar‐Shir , Y. Liang , K. W. Y. Chan , A. A. Gilad , J. W. M. Bulte , Chem. Commun. 2015, 51, 4869. 10.1039/c4cc10195b PMC4485562 25697683

[38] M. T. McMahon , A. A. Gilad , M. A. DeLiso , S. M. C. Berman , J. W. M. Bulte , P. C. M. van Zijl , Magn. Reson. Med. 2008, 60, 803. 18816830 10.1002/mrm.21683 PMC2614370

[39] M. T. McMahon , A. A. Gilad , J. Zhou , P. Z. Sun , J. W. M. Bulte , P. C. M. van Zijl , Magn. Reson. Med. 2006, 55, 836. 16506187 10.1002/mrm.20818 PMC2860536

[40] J. Zhou , J.‐F. Payen , D. A. Wilson , R. J. Traystman , P. C. M. van Zijl , Nat. Med. 2003, 9, 1085. 12872167 10.1038/nm907

[41] C. T. Farrar , J. S. Buhrman , G. Liu , A. Kleijn , M. L. M. Lamfers , M. T. McMahon , A. A. Gilad , G. Fulci , Radiology 2015, 275, 746. 25686366 10.1148/radiol.14140251 PMC4450912

[42] I. Minn , A. Bar‐Shir , K. Yarlagadda , J. W. M. Bulte , P. B. Fisher , H. Wang , A. A. Gilad , M. G. Pomper , Magn. Reson. Med. 2015, 74, 544. 25919119 10.1002/mrm.25748 PMC4612624

[43] S. Meier , A. A. Gilad , J. A. Brandon , C. Qian , E. Gao , J. F. Abisambra , M. Vandsburger , Sci. Rep. 2018, 8, 4638. 29545551 10.1038/s41598-018-22993-4 PMC5854573

[44] O. Perlman , H. Ito , A. A. Gilad , M. T. McMahon , E. A. Chiocca , H. Nakashima , C. T. Farrar , Sci. Rep. 2020, 10, 20664. 33244130 10.1038/s41598-020-77576-z PMC7692519

[45] A. R. Bricco , I. Miralavy , S. Bo , O. Perlman , D. E. Korenchan , C. T. Farrar , M. T. McMahon , W. Banzhaf , A. A. Gilad , ACS Synth. Biol. 2023, 12, 1154. 36947694 10.1021/acssynbio.2c00648 PMC10128068

[46] A. J. Fillion , A. R. Bricco , H. D. Lee , D. E. Korenchan , C. T. Farrar , A. A. Gilad , NMR Biomed. 2023, 36, e5007. 37469121 10.1002/nbm.5007 PMC11075521

[47] N. Scalzitti , I. Miralavy , D. E. Korenchan , C. T. Farrar , A. A. Gilad , W. Banzhaf , J. Comput. Aided Mol. Des. 2024, 38, 17. 38570405 10.1007/s10822-024-00558-0 PMC11416381

[48] O. Perlman , H. Ito , K. Herz , N. Shono , H. Nakashima , M. Zaiss , E. A. Chiocca , O. Cohen , M. S. Rosen , C. T. Farrar , Nat. Biomed. Eng. 2021, 6, 648. 34764440 10.1038/s41551-021-00809-7 PMC9091056

[49] S. Waelder , L. Lee , A. G. Redfield , J. Am. Chem. Soc. 1975, 97, 2927. 1133343 10.1021/ja00843a066

[50] S. F. Waelder , A. G. Redfield , Biopolymers 1977, 16, 623. 14742 10.1002/bip.1977.360160311

[51] T. Takahashi , M. Nakanishi , M. Tsuboi , Anal. Biochem. 1981, 110, 242. 7212267 10.1016/0003-2697(81)90142-1

[52] Y. Kawata , Y. Goto , K. Hamaguchi , F. Hayashi , Y. Kobayashi , Y. Kyogoku , Biochemistry 1988, 27, 346. 2831958 10.1021/bi00401a052

[53] J. D. J. O'Neil , B. D. Sykes , Biochemistry 1989, 28, 6736. 2790027 10.1021/bi00442a029

[54] G. Otting , E. Liepinsh , K. Wuethrich , J. Am. Chem. Soc. 1991, 113, 4363.

[55] E. Liepinsh , G. Otting , K. Wüthrich , J. Biomol. NMR 1992, 2, 447. 1384851 10.1007/BF02192808

[56] E. Liepinsh , G. Otting , Magn. Reson. Med. 1996, 35, 30. 8771020 10.1002/mrm.1910350106

[57] X. Yang , X. Song , Y. Li , G. Liu , S. Ray Banerjee , M. G. Pomper , M. T. McMahon , Angew. Chem. Int. Ed. 2013, 52, 8116. 10.1002/anie.201302764 PMC3819166 23794432

[58] X. Yang , N. N. Yadav , X. Song , S. Ray Banerjee , H. Edelman , I. Minn , P. C. M. van Zijl , M. G. Pomper , M. T. McMahon , Chem. ‐ Eur. J. 2014, 20, 15824. 25302635 10.1002/chem.201403943 PMC4309366

[59] R. T. Oglesby , W. W. Lam , G. J. Stanisz , Magn. Reson. iMed. 2020, 84, 2389. 10.1002/mrm.28281 32301165

[60] M. Zaiss , P. Bachert , Phys. Med. Biol. 2013, 58, R221. 24201125 10.1088/0031-9155/58/22/R221

[61] T. Jin , J. Autio , T. Obata , S.‐G. Kim , Magn. Reson. Med. 2011, 65, 1448. 21500270 10.1002/mrm.22721 PMC3061975

[62] J. P. Gallivan , D. A. Dougherty , Proc. Natl. Acad. Sci. USA 1999, 96, 9459. 10449714 10.1073/pnas.96.17.9459 PMC22230

[63] L. M. Firsov , K. N. Neustroev , A. E. Aleshin , C. M. Metzler , D. E. Metzler , R. D. Scott , B. Stoffer , T. Christensen , B. Svensson , Eur. J. Biochem. 1994, 223, 293. 8033904 10.1111/j.1432-1033.1994.tb18994.x

[64] S. Goerke , K. S. Milde , R. Bukowiecki , P. Kunz , K. D. Klika , T. Wiglenda , A. Mogk , E. E. Wanker , B. Bukau , M. E. Ladd , P. Bachert , M. Zaiss , NMR Biomed. 2017, 30, e3665. 10.1002/nbm.3665 27859838

[65] S. Goerke , M. Zaiss , P. Kunz , K. D. Klika , J. D. Windschuh , A. Mogk , B. Bukau , M. E. Ladd , P. Bachert , NMR Biomed. 2015, 28, 906. 26010522 10.1002/nbm.3317

[66] S. Goerke , Y. Soehngen , A. Deshmane , M. Zaiss , J. Breitling , P. S. Boyd , K. Herz , F. Zimmermann , K. D. Klika , H. Schlemmer , D. Paech , M. E. Ladd , P. Bachert , Magn. Reson. Med. 2019, 82, 622. 30927313 10.1002/mrm.27751

[67] C. B. Lauzon , P. Van Zijl , J. T. Stivers , J. Biomol. NMR 2011, 50, 299. 21809183 10.1007/s10858-011-9527-z PMC3149851

[68] R. A. Kumpf , D. A. Dougherty , Science 1993, 261, 1708. 8378771 10.1126/science.8378771

[69] S. Feske , H. Wulff , E. Y. Skolnik , Annu. Rev. Immunol. 2015, 33, 291. 25861976 10.1146/annurev-immunol-032414-112212 PMC4822408

[70] T. H. Hansen , M. Bouvier , Nat. Rev. Immunol. 2009, 9, 503. 19498380 10.1038/nri2575

[71] P. A. Roche , K. Furuta , Nat. Rev. Immunol. 2015, 15, 203. 25720354 10.1038/nri3818 PMC6314495

[72] T. R. Eykyn , G. S. Payne , M. O. Leach , Phys. Med. Biol. 2005, 50, N371. 16264247 10.1088/0031-9155/50/22/N03

[73] X. Xu , J.‐S. Lee , A. Jerschow , Angew. Chem., Int. Ed. 2013, 52, 8281. 10.1002/anie.201303255 PMC3827864 23813633

[74] L. Zhang , Y. Zhao , Y. Chen , C. Bie , Y. Liang , X. He , X. Song , Quant. Imaging Med. Surg. 2019, 9, 1714. 31728314 10.21037/qims.2019.10.01 PMC6828582

[75] M. Zaiss , G. Angelovski , E. Demetriou , M. T. McMahon , X. Golay , K. Scheffler , Magn. Reson. Med. 2018, 79, 1708. 28686796 10.1002/mrm.26813

[76] J. Rey , S. Murail , S. de Vries , P. Derreumaux , P. Tuffery , Nucleic Acids Res. 2023, 51, W432. 37166962 10.1093/nar/gkad376 PMC10320157

[77] P. Tufféry , P. Derreumaux , Front. Bioinform. 2023, 3, 1113928. 36727106 10.3389/fbinf.2023.1113928 PMC9885153

[78] V. Binette , N. Mousseau , P. Tuffery , J. Chem. Theory Comput. 2022, 18, 2720. 35298162 10.1021/acs.jctc.1c01293

[79] A. Lamiable , P. Thévenet , J. Rey , M. Vavrusa , P. Derreumaux , P. Tufféry , Nucleic Acids Res. 2016, 44, W449. 27131374 10.1093/nar/gkw329 PMC4987898

[80] M. Kim , J. Gillen , B. A. Landman , J. Zhou , P. C. M. van Zijl , Magn. Reson. Med. 2009, 61, 1441. 19358232 10.1002/mrm.21873 PMC2860191

[81] M. Gram , M. Seethaler , D. Gensler , J. Oberberger , P. M. Jakob , P. Nordbeck , Magn. Reson. iMed. 2021, 85, 2771. 10.1002/mrm.28585 33166009

[82] H. Nakashima , T. Nguyen , K. Kasai , C. Passaro , H. Ito , W. F. Goins , I. Shaikh , R. Erdelyi , R. Nishihara , I. Nakano , D. A. Reardon , A. C. Anderson , V. Kuchroo , E. A. Chiocca , Clin. Cancer Res. 2018, 24, 2574. 29511029 10.1158/1078-0432.CCR-17-2954 PMC6800093

[83] V. Khlebnikov , W. J. M. van der Kemp , H. Hoogduin , D. W. J. Klomp , J. J. Prompers , Sci. Rep. 2019, 9, 1089. 30705355 10.1038/s41598-018-37295-y PMC6355971

