# Supplementary material for: Optimization of CEST MRI Reporter Protein Design Using Cation‐Pi Networks
Source: Chemistry. 2025 Sep 10;32(28):e01638. doi: 10.1002/chem.202501638 (PMC13411488; doi:10.1002/chem.202501638)
Supplement: Supplementary file 1 — Supporting Information [file CHEM-32-e01638-s001.docx]

Supporting Information for:

Optimization of CEST MRI Reporter Protein Design Using Cation-Pi Networks

David E. Korenchan,^[a]^ Ethan J. French, ^§[a]^ Emerenziana Runco, ^§[b]^ Chetan B. Dhakan, ^[a]^ Jinwu Yan, ^[a]^ Hiroshi Nakashima,^[b]^ Michael T. McMahon,^[c,d]^ Assaf A. Gilad,^[e,f]^ and Christian T. Farrar^*[a]^

^[a]^ *Athinoula A. Martinos Center for Biomedical Imaging, Massachusetts General Hospital, 149 13th Street, Room 2301, Charlestown, MA 02129, United States*
^[b]^ *Department of Neurosurgery, Harvey Cushing Neuro-oncology Laboratories, Brigham and Women’s Hospital, Boston, MA 02115, United States*

^[c]^ *F.M. Kirby Research Center for Functional Brain Imaging, Kennedy Krieger Institute, Baltimore, MD 21205, United States*

^[d]^ *Department of Radiology, Johns Hopkins School of Medicine, Baltimore, MD 21287, United States*

^[e]^ *Department of Chemical Engineering and Materials Science, Michigan State University, East Lansing, MI 48824, United States*

^[f]^ *Department of Radiology, Michigan State University, East Lansing, MI 48824, United States*

^§^ These authors contributed equally.

* Email: cfarrar@mgh.harvard.edu

Table of Contents

[Supporting Methods 3](#_Toc207287497)

[Chemical reagents and sample preparation 3](#_Toc207287498)

[^1^H NMR spectroscopy and CEST measurements 3](#_Toc207287499)

[Circular dichroism (CD) spectroscopy 4](#_Toc207287500)

[Fluorescence spectroscopy 4](#_Toc207287501)

[In vitro CEST imaging 4](#_Toc207287502)

[NMR data processing 5](#_Toc207287503)

[Processing of in vitro CEST imaging data 7](#_Toc207287504)

[Computational peptide structure prediction and display 7](#_Toc207287505)

[In vivo CEST imaging in mouse brain 8](#_Toc207287506)

[Processing of in vivo CEST imaging data 8](#_Toc207287507)

[Results and Discussion 9](#_Toc207287508)

[BPTI CEST spectral fitting to measure Tyr proton exchange rates over varying temperatures and pH 9](#_Toc207287509)

[QUESP analysis of proton pool exchange in all Trp-containing peptides 11](#_Toc207287510)

[QUESP analysis of proton pool exchange in peptide KMWDWQKKKWI 14](#_Toc207287511)

[CEST NMR investigation of other amino acid substitution effects on the 4.2 ppm signal 15](#_Toc207287512)

[Peptide structure simulation with PEP-FOLD4 16](#_Toc207287513)

[CD spectroscopy of short peptides 17](#_Toc207287514)

[Fluorescence spectroscopy of short peptides 18](#_Toc207287515)

[Spectral fitting and QUESP analysis of glucoamylase CEST spectra 19](#_Toc207287516)

[In vitro CEST and CESL spectroscopic imaging of glucoamylase and poly-L-lysine 20](#_Toc207287517)

[In vivo CEST spectroscopic imaging in mouse brain 23](#_Toc207287518)

[Peptide Characterization 24](#_Toc207287519)

[KMWDWEQKKKWI, HPLC 24](#_Toc207287520)

[KMWDWEQKKKWI, Mass Spectrometry 25](#_Toc207287521)

[KMWDWQKKKWI, HPLC 26](#_Toc207287522)

[KMWDWQKKKWI, Mass Spectrometry 28](#_Toc207287523)

[OMWDWQOOOWI, HPLC 29](#_Toc207287524)

[OMWDWQOOOWI, Mass Spectrometry 31](#_Toc207287525)

[KMWDWQKOOWI, HPLC 32](#_Toc207287526)

[KMWDWQKOOWI, Mass Spectrometry 33](#_Toc207287527)

[KM[5-HTP]DWQKKKWI, HPLC 34](#_Toc207287528)

[KM[5-HTP]DWQKKKWI, Mass Spectrometry 35](#_Toc207287529)

[KMWDWQKKK[5-HTP]I, HPLC 36](#_Toc207287530)

[KMWDWQKKK[5-HTP]I, Mass Spectrometry 38](#_Toc207287531)

[OMWDWQKOOWI, HPLC 39](#_Toc207287532)

[OMWDWQKOOWI, Mass Spectrometry 40](#_Toc207287533)

[OMWDWQKOKWI, HPLC 41](#_Toc207287534)

[OMWDWQKOKWI, Mass Spectrometry 43](#_Toc207287535)

[Supporting References 44](#_Toc207287536)

# Supporting Methods

This section includes more detailed descriptions of the methods in the main manuscript, plus methods relevant to the figures in the Supporting Information. Please note that no unexpected or unusually high safety hazards were encountered in any experiments performed in this work.

## Chemical reagents and sample preparation

All synthetic peptides were obtained from GenScript (Piscataway, NJ, USA). Bovine pancreatic trypsin inhibitor (BPTI; also known as aprotinin) was purchased from Sigma Aldrich (cat #A1153, St. Louis, MO, USA). Glucoamylase from *Rhizopus sp.* was purchased from Sigma-Aldrich (cat #172426). Poly-L-lysine hydrobromide (PLL, 4-15 kDa molecular weight) was purchased from Sigma-Aldrich (cat #P6516). For peptide NMR sample preparation, 4-5 mg of each peptide was resuspended in 600 μL of 1x phosphate-buffered saline (PBS; 137 mM NaCl, 2.7 mM KCl, 11.9 mM phosphate) along with 1 mM of sodium formate, used as a concentration reference. The BPTI samples were prepared by dissolving 5-7 mg of lyophilized protein in 600 μL of 1x PBS and titrating to the desired pH. The glucoamylase NMR spectroscopy sample was prepared by dissolving 34.5 mg of lyophilized protein in 600 μL of 1x PBS, and the glucoamylase CEST imaging sample was prepared by dissolving 35.3 mg of lyophilized protein in 725 μL of 1x PBS. The PLL sample was prepared by dissolving 5.0 mg of lyophilized protein in 700 μL of 1x PBS. For all samples but the BPTI, the pH was adjusted to 7.30 ± 0.05 at room temperature by titrating with either HCl or NaOH and using a pH meter (Orion PerpHect Meter, Model 330, Thermo Scientific, Waltham, MA, USA). The pH meter temperature was set to 23 °C and was calibrated using pH 7.00 and 10.01 standard buffers (Thermo Scientific). The sample was then pipetted into a 5 mm borosilicate glass NMR tube.

## ^1^H NMR spectroscopy and CEST measurements

All samples were measured using a 14 T (600 MHz) Bruker NMR spectrometer equipped with an indirect-detect, dual-tuned ^1^H/broadband probe and running Topspin software (Bruker Corporation, Billerica, MA, USA). The temperature was set to 37 °C, and each sample was allowed to equilibrate for at least 40 minutes prior to the start of CEST z-spectroscopy. For each sample, the 90° pulse width was calibrated using the 360° zero-crossing, a water-suppressed ^1^H NMR spectrum was acquired, and the spin-lattice relaxation time constant (*T*_1_) was measured using an inversion-recovery pulse sequence with the z-gradient on at 5% amplitude between the inversion and excitation pulses, to minimize radiation damping.^[1]^ The acquisition parameters for these experiments were generally the following: 16384 complex FID points acquired, receiver bandwidth = 7.2 kHz, and the total repetition time set to at least 5**T*_1,water_. CEST z-spectroscopy was performed using an ultrafast z-spectroscopy sequence^[2]^ and the following parameters: 1024 complex FID points acquired, 42.6 kHz receiver bandwidth, 32 or 64 scans, 10 s recovery delay between scans, 5% gradient applied during saturation and acquisition, pulse offset frequency set to be about 3250 Hz higher than water frequency, 5 s saturation pulse, and saturation pulse attenuation values of [40, 38, 36, 35, 34, 33, 32, 31, 30, 28] dB, which generally translated to amplitudes of 1.2-5.7 μT (350-1500 rad s^-1^), depending on the coil loading. Most of the Trp-containing peptides were measured without the three highest saturation amplitudes and, therefore, with a maximum amplitude of ~3.2 μT (~850 rad s^-1^). Four dummy scans were performed between each saturation power value. Each sample also included two reference scans, one at the beginning and one at the end, during which the saturation amplitude was set to 0. These were used to calculate CEST z-spectra and ensure sample stability. For most samples, the probe was tuned and matched prior to all sequences; however, samples that were run at multiple temperatures (such as the BPTI and glucoamylase samples) were tuned and matched at one temperature, then pulses were recalibrated at subsequent temperatures using the procedure described above. This calibrated 90° pulse value was used for subsequent calculations of the saturation amplitudes.

## Circular dichroism (CD) spectroscopy

CD spectroscopy was performed with a JASCO J-1500 CD spectrometer (JASCO, Tokyo, Japan). Each of the peptide samples prepared for CEST NMR spectroscopy was diluted by a factor of 60-80 in 12 mM phosphate buffer, pH 7.3. 180 µL of solution was then pipetted into a cuvette with a 1 mm path length for spectroscopy. Scanning parameters included a digital integration time of 4 s, a scanning speed of 50 nm/min, and measurement wavelengths from 260 to 190 nm in 0.5 nm increments.

## Fluorescence spectroscopy

Fluorescence spectroscopy was performed using a Hitachi F-4500 fluorescence spectrophotometer (Hitachi, Chiyoda, Tokyo, Japan). Each peptide sample used for CEST NMR spectroscopy was diluted by a factor of 2 using 1x PBS prior to loading into the cuvette for measurement. The excitation and emission wavelengths used were 280 nm and 290-500 nm, respectively. The scan speed was 2400 nm/min, the emission wavelength resolution was 0.2 nm, and the excitation and emission slit widths were 5 nm each.

## In vitro CEST imaging

Imaging was performed on a 9.4T Bruker small-bore scanner running Paravision 360 software (version 3.5) and equipped with a quadrature ^1^H probe (86 mm inner diameter). Three 700 μL tubes containing the imaging samples were placed within a 50 mL conical tube, which was filled with water and placed within the scanner surrounded by a 37 °C isothermal heat pack. Following positioning, the coils were tuned and matched, the transmit/receive frequency and excitation pulse was calibrated, and localized shimming was performed on a cuboid region encompassing all three tubes. A single-shot echo-planar imaging (EPI) sequence (5 mm slice thickness, 30x30 mm^2^ imaging field of view, 64x64 matrix size) was performed at the following 52 saturation offsets, alternating positive and negative offsets: +1247 ppm (unsaturated image); ±8 to ±6.6 ppm in increments of 0.2 ppm (also including 7.3 ppm); ±6 to ±4 ppm in increments of 0.5 ppm; ±4 to ±3 ppm in increments of 0.2 ppm (also including 3.5 ppm); ±3 to 0 ppm in increments of 0.5 ppm. The saturation duration (*T*_sat_) was set to 10 s, and the repetition time (TR) was set to 20 s. The imaging was repeated for five saturation amplitudes: [2,3,5,7,9] μT. The measured *T*_1_ of water using a saturation-recovery sequence was ≤4 s in each tube, so the saturation duration was enough to nearly reach steady state (i.e. *T*_sat_ ≥ 2.5 * *T*_1_). A WAter Saturation Shift Referencing (WASSR) sequence^[3]^ was also performed (-0.5 to 0.5 ppm in 0.05 ppm increments, alternating positive and negative frequencies) to measure the *B*_0_ inhomogeneity profile.

Additionally, chemical exchange spin-locking (CESL) imaging was performed with identical imaging parameters as the CEST imaging. In these experiments, a 90° pulse was used to tip the water resonance into the xy-plane, then a balanced spin-lock pulsing scheme^[4]^ parallel with the excited water magnetization was applied for a duration *T*_lock_. The remaining water magnetization was then returned to the z-axis with another 90° pulse with opposite the phase of the first 90° pulse, and then the water was excited and imaged just as in the CEST imaging experiments. The spin-lock pulse amplitude was varied just as the CEST saturation amplitude, with amplitudes of [2,3,5,7,9] μT. The locking duration *T*_lock_ was varied as well, with 12 values: [50,100,150,200,300,400,500,750,1000,2000,3000,5000] ms.

## NMR data processing

Conventional ^1^H NMR data, including the inversion-recovery sequence, were processed using Bruker TopSpin software. Ultrafast z-spectroscopy CEST data were analyzed using custom scripts written in MATLAB (MathWorks, Natick, MA, USA). Raw data were apodized exponentially by 100 Hz and zero-filled by a factor of 16. All raw spectra were normalized to have the same value at 12 ppm downfield of the water resonance (for glucoamylase, 15 ppm was used due to the larger ^1^H chemical shifts encountered), and then z-spectra across all saturation amplitudes were calculated by dividing by the spectral profile obtained without saturation. The magnetization transfer ratio asymmetry (*MTR*_asym_) was calculated using the following equation:

$${MTR}_{asym}=Z\left( -\delta\right)-Z\left( \delta\right)=\frac{S\left( -\delta\right)}{S_{ref}\left( -\delta\right)}-\frac{S\left( \delta\right)}{S_{ref}\left( \delta\right)}$$

Here, $S\left( \delta\right)$ and $S\left( -\delta\right)$ refer to the signal magnitude on opposite sides of the water resonance at a chemical shift of magnitude $\delta$, and $S_{ref}$ is the signal profile obtained without saturation. The CEST MTR spectral profiles were also fitted using Pseudo-Voigt lineshapes^[5]^ to isolate the contributions to the z-spectra from various exchanging proton pools, including hydroxyl, amine, amide, and Trp indole NH. More details on the spectral profile fitting can be found in the Supporting Information. Using the fitted Pseudo-Voigt lineshape amplitudes, the proton volume fraction (*f*_s_) and exchange rate (*k*_sw_) for each pool were then determined with QUantification of Exchange rate using varying Saturation Power (QUESP),^[6,7]^ specifically employing Equation [12] of reference^[7]^ and correcting the initial magnetization *Z*_i_ using the measured water *T*_1_ value and the relaxation delay. The saturation amplitudes (in units of rad/s) were calculated from the power attenuation values (in dB) using the 90° pulse width calibration. All MATLAB code used to process the CEST spectral data is accessible on GitHub (<https://github.com/dkorenchan/ultrafastZspecCEST_sequence_processing>).

*Pseudo-Voigt peak fitting of CEST MTR spectra*

Each Pseudo-Voigt lineshape was calculated as:

$MTR(\omega)=1-Z\left( \omega\right)=1-\frac{S\left( \omega\right)}{S_{ref}\left( \omega\right)}$ (S1)

As in the main text, $S\left( \omega\right)$ refers to the signal magnitude at a chemical shift of magnitude $\omega$ from the water resonance, and $S_{ref}$ is the signal profile obtained without saturation. Each Pseudo-Voigt lineshape $\mathcal{V}\left( \omega\right)$ was calculated as the weighted sum of a Lorentzian lineshape $\mathcal{L}\left( \omega\right)$ with full-width at half-maximum $\Gamma$ and a Gaussian lineshape $\mathcal{G}\left( \omega\right)$ with a linewidth parameter $\sigma$:

$\mathcal{V}\left( \omega\right)=A\left( \chi\mathcal{L}\left( \omega\right)+\left( 1-\chi\right)\mathcal{G}\left( \omega\right) \right)$ (S2)

$\mathcal{L}\left( \omega\right)=\frac{\left( \frac{\Gamma}{2} \right)^{2}}{\left( \omega-\omega_{off} \right)^{2}+\left( \frac{\Gamma}{2} \right)^{2}}$ (S3)

$\mathcal{G}\left( \omega\right)=e^{-\frac{\left( \omega-\omega_{off} \right)^{2}}{2\sigma^{2}}}$ (S4)

$\sigma=\alpha\frac{\Gamma}{2\sqrt{2\ln\left( 2 \right)}}$ (S5)

Thus, for each lineshape there were five parameters fitted: the amplitude $A$, the peak offset $\omega_{off}$, the Lorentzian linewidth $\Gamma$, the scaling factor $\alpha$ between the Lorentzian and Gaussian linewidth parameters (constrained such that $1\leq\alpha\leq2$), and the Lorentzian proportion $\chi$. Note that both expressions for $\mathcal{L}\left( \omega\right)$ and $\mathcal{G}\left( \omega\right)$ were normalized to have a *maximum amplitude* of 1, rather than an *integral* of 1.

All datasets included fitting for the exchangeable protons pertaining to the hydroxyl (0.6-1.0 ppm) and amine (1.3-2.3 ppm) pools. All but the BPTI data were fitted for the amide (3.0-4.0 ppm) pool as well. Additional fitted pools included Trp indole NH (5.3-5.7 ppm), Tyr phenol OH/4.2 ppm (4.0-5.0 ppm), and pools at 7.3 ppm (7.0-8.0 ppm), and 9.8 ppm (9.0-11.0 ppm). For all datasets, the water peak was fit first using only the negative ppm values $-5\leq\omega\leq0$ (since generally little to no macromolecular magnetization transfer (MT) or nuclear Overhauser effect (NOE) were observed in this range), and then all other peaks were fit while fixing the water pool fitting parameters. For most datasets, the peaks were first fit for the second-lowest saturation amplitude, and then the fitted peak offsets ($\omega_{off,i}$) were used for fitting the remaining spectra. Furthermore, the fitting constrained the Pseudo-Voigt peak character (i.e. the values of $\alpha$ and $\chi$) to be equal across all CEST peaks except water at each saturation amplitude. For the BPTI and glucoamylase datasets, the fitted peak offsets were determined using the sixth-lowest saturation amplitude. For the glucoamylase dataset, the Pseudo-Voigt character was unconstrained across all peaks.

All MATLAB code used to process the CEST spectral data is accessible on GitHub (<https://github.com/dkorenchan/ultrafastZspecCEST_sequence_processing>).

## Processing of in vitro CEST imaging data

All *in vitro* imaging data were processed using custom MATLAB scripts, available on GitHub (<https://github.com/dkorenchan/BrukerCESTmethod_processingPipeline>). The imaging data were processed by adjusting the z-spectral profile based upon the input WASSR shift for each voxel. Imaging voxels with signal-to-noise ratio (SNR) below 3.5 in the unsaturated image were removed from the analysis. The *MTR*_asym_ was calculated on a per-voxel basis using the same equation as for the ultrafast z-spectroscopy data. Following image processing, a region of interest (ROI) was drawn on each tube, and the *MTR*_asym_ mean and standard deviation of the encompassed voxels were calculated.

## Computational peptide structure prediction and display

Peptide structure prediction was performed using the PEP-FOLD4 algorithm,^[8–11]^ available online at <https://mobyle2.rpbs.univ-paris-diderot.fr/cgi-bin/portal.py>. The following simulation parameters were used to generate the lowest-energy conformations for the KMWDWEQKKKWI and KMWDWQKKKWI peptide sequences: ts3 generator, 200 independent models run, 30,000 Monte-Carlo steps with a temperature of 370 K, a pseudo-random seed set to 1, Debye-Hückel contribution included, pH 7.5, ionic strength 150 mM (expressed as equivalents of NaCl), and no blocking of extremities (i.e. N- and C-termini). The resulting .pdb structure files were downloaded and visualized using PyMOL software (Schrödinger, New York, NY, USA).

## In vivo CEST imaging in mouse brain

All mouse imaging was performed in accordance with animal imaging protocols established by the Institutional Animal Care & Use Committee (IACUC) at Massachusetts General Hospital/Brigham Women’s Hospital (protocol #2016N000383). A BL6 MacGreen transgenic mouse was implanted stereotactically with 100,000 GL261N4 cells in the right frontal lobe (2 mm anterior to and 0.5 mm to the right of the bregma, then 3.5 mm inferior) of the brain on Day 0, as previously described.^[12,13]^ On Day 18, the mouse was anesthetized, secured in a mouse holder with anesthesia delivered through a nose cone, and placed within a 7T Bruker horizontal-bore preclinical scanner. Imaging was performed with a ^1^H volume coil for transmit and a 30 mm diameter ^1^H surface coil for receive. Following coil tuning and matching, frequency and power calibrations, the mouse was imaged with a multi-slice multi-echo (MSME) anatomical imaging sequence, and then a CEST spin-echo echo-planar imaging (SE-EPI) sequence was run at two saturation amplitudes: 0.7 and 3.5 μT. The imaging parameters were the following: 64x64 imaging matrix size, 19x19 mm^2^ field of view, 297x297 μm^2^ in-plane spatial resolution, 1 mm slice thickness, 8 s TR, 20 ms TE, 3 s continuous-wave saturation, saturation offsets from -9 to +9 ppm in 0.25 ppm increments, 1 average, total imaging time 9m52s. The CEST imaging also included one saturation offset that was far off-resonance (1 MHz) which was used for calculating z-spectra. After imaging, the mouse was removed from the scanner and allowed to recover. Note that no visible tumor region was observed in the mouse brain.

## Processing of in vivo CEST imaging data

The mouse brain CEST data were processed using a custom Python script by drawing a region of interest over the entire brain, averaging the signal across the voxels for each saturation offset, and then dividing the signal by that obtained with the far off-resonance saturation to calculate z-spectra. A Savitzky-Golay smoothing filter was applied over the z-spectrum with a window length of 8 and a polynomial fitting order of 3 using the scipy.signal.savgol_filter() function in Python.

# Results and Discussion

##

## BPTI CEST spectral fitting to measure Tyr proton exchange rates over varying temperatures and pH

**Figure S1** shows the MTR asymmetry plots obtained for BPTI as a function of pH (6.6-7.3) and temperature (10-37 °C). The Tyr OH CEST peak at ~4.8 ppm tended to broaden out at the highest temperature (37 °C), as well as at the lowest temperature and pH (pH 6.6, 10 °C). However, the phenol OH exchange rate did not change significantly enough to suggest that this was exchange broadening: *k*_sw_ remained between 1250 and 1560 s^-1^ between 10-37 ºC and pH 6.6-7.3 (see **Table S1**).


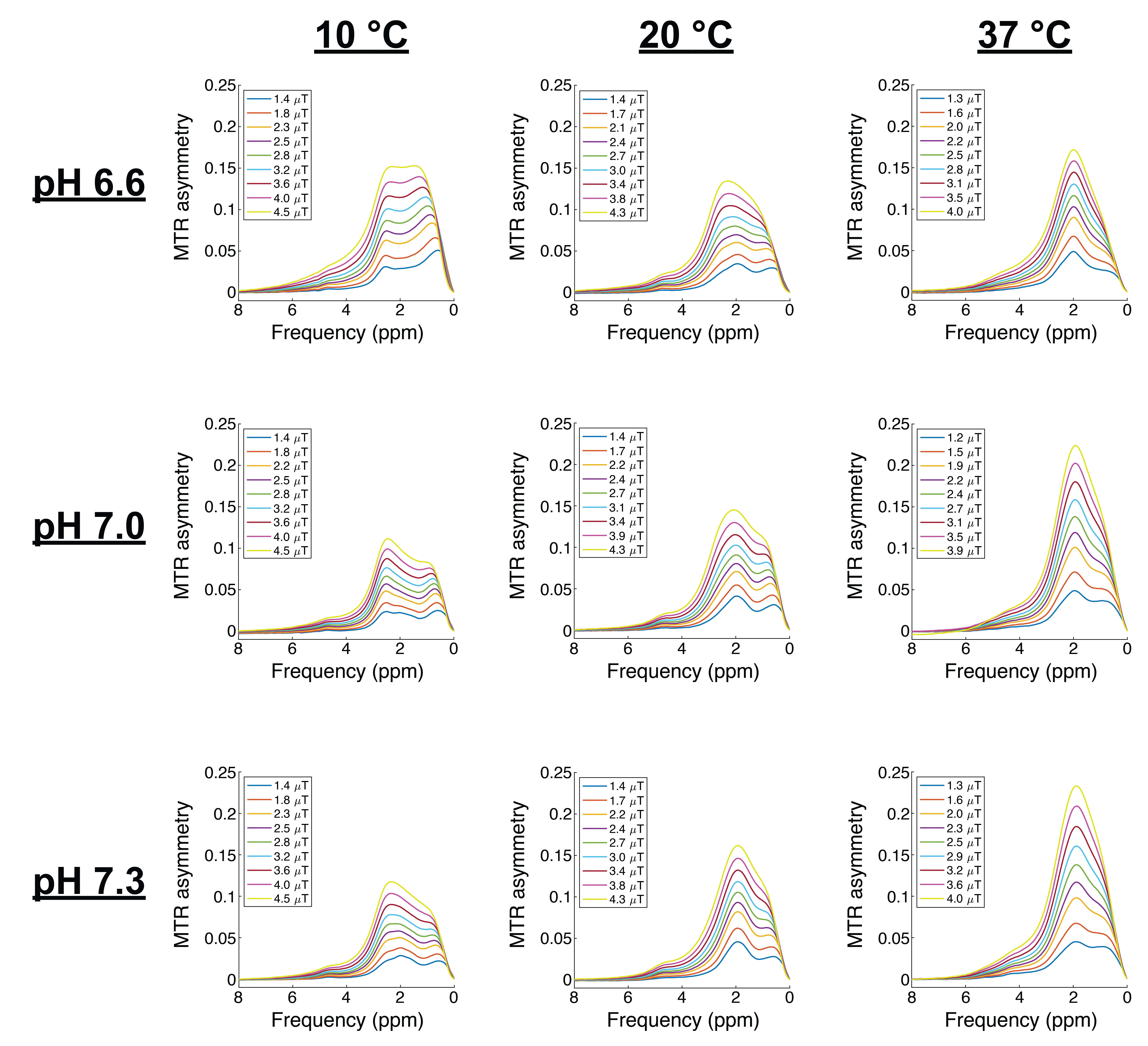


**Figure S1*.*** CEST MTR asymmetry plots for BPTI prepared and measured at different pH and temperature values. The protein concentration was 9.33-11.17 mg/mL. Note that only the lowest 9 saturation amplitudes are shown for each dataset; some of the datasets included an additional 10^th^ saturation amplitude.

The fitted parameters for the Tyr phenol pool and amine/amide pool (resonating between 2-2.6 ppm) are summarized in **Table S1** below. Because of the broad nature of the Tyr phenol pool, it was difficult to properly fit this peak in the CEST spectra, especially at the highest temperature measured (37 °C). This may be due to multiple Tyr residues contributing to the signal, each with a slightly different chemical shift and with varying contributions as both temperature and pH change.^[14]^ The Tyr phenol exchange rate remained within a fairly narrow range, between 1260 and 1560 s^-1^, for pH values 6.6-7.3 and temperatures 10-37 °C.

**Table S1*.*** Proton exchange rates obtained from BPTI samples using spectral fitting and QUESP analysis. Note that 10 saturation amplitudes were used for spectral fitting and QUESP for all but three datasets: the [pH 6.6, 20 °C] and [pH 6.6, 37 °C] datasets were acquired without the highest saturation amplitude, and the [pH 6.6, 10 °C] dataset omitted the highest three saturation amplitudes due to poor fitting of the CEST spectra at these high saturation amplitudes.

| **pH** | **Temperature (°C)** | *Amine NH_3_/amide NH* | | | | |
| --- | --- | --- | --- | --- | --- | --- |
|  |  | ***f*_s_** | ***f*_s_ fit error** | ***k*_sw_ (s^-1^)** | ***k*_sw_ fit error (s^-1^)** | ***R*^2^** |
| 6.6 | 10 | 231 x 10^-6^ | 14 x 10^-6^ | 1806 | 164 | 1.000 |
| 6.6 | 20 | 154 x 10^-6^ | 4.7 x 10^-6^ | 1275 | 100 | 0.999 |
| 6.6 | 37 | 176 x 10^-6^ | 1.2 x 10^-6^ | 829 | 22 | 1.000 |
| 7.0 | 10 | 108 x 10^-6^ | 3.5 x 10^-6^ | 1252 | 131 | 0.994 |
| 7.0 | 20 | 129 x 10^-6^ | 6.0 x 10^-6^ | 1209 | 180 | 0.988 |
| 7.0 | 37 | 186 x 10^-6^ | 0.7 x 10^-6^ | 1096 | 12 | 1.000 |
| 7.3 | 10 | 118 x 10^-6^ | 4.1 x 10^-6^ | 1334 | 141 | 0.995 |
| 7.3 | 20 | 142 x 10^-6^ | 5.2 x 10^-6^ | 1063 | 133 | 0.990 |
| 7.3 | 37 | 190 x 10^-6^ | 2.4 x 10^-6^ | 1303 | 44 | 1.000 |

| **pH** | **Temperature (°C)** | *Tyr phenol OH* | | | | |
| --- | --- | --- | --- | --- | --- | --- |
|  |  | ***f*_s_** | ***f*_s_ fit error** | ***k*_sw_ (s^-1^)** | ***k*_sw_ fit error (s^-1^)** | ***R*^2^** |
| 6.6 | 10 | 32.0 x 10^-6^ | 8.3 x 10^-6^ | 1559 | 697 | 0.987 |
| 6.6 | 20 | 17.5 x 10^-6^ | 1.6 x 10^-6^ | 1399 | 309 | 0.990 |
| 6.6 | 37 | 29.4 x 10^-6^ | 0.4 x 10^-6^ | 1267 | 37 | 1.000 |
| 7.0 | 10 | 14.9 x 10^-6^ | 1.2 x 10^-6^ | 1426 | 334 | 0.975 |
| 7.0 | 20 | 15.5 x 10^-6^ | 0.9 x 10^-6^ | 1332 | 244 | 0.984 |
| 7.0 | 37 | 26.5 x 10^-6^ | 1.0 x 10^-6^ | 1426 | 134 | 0.997 |
| 7.3 | 10 | 14.7 x 10^-6^ | 1.0 x 10^-6^ | 1445 | 286 | 0.983 |
| 7.3 | 20 | 21.4 x 10^-6^ | 0.7 x 10^-6^ | 1440 | 127 | 0.997 |
| 7.3 | 37 | 27.1 x 10^-6^ | 0.8 x 10^-6^ | 1326 | 109 | 0.997 |

##

## QUESP analysis of proton pool exchange in all Trp-containing peptides

**Table S2** summarizes the volume fractions and exchange rate constants of the amine, amide, and Trp indole NH protons measured in the Trp-containing peptides. An example of the CEST spectrum and QUESP fitting for one peptide is shown in **Figure S2**. For several peptides, the amine fitting had a high fit error, likely due to the rapid exchange rate relative to the maximum saturation amplitude. Several peptides also had a high *k*_sw_ fit error for the Trp indole NH pool, perhaps because of the larger, broader amide CEST peak interfering with the pool fitting. Nevertheless, all but 1 peptide had a good coefficient of determination (*R*^2^ > 0.95) for the QUESP fits.

For the sequences with three Trp residues (12 of 15 peptides), the mean ± s.d. amide:indole proton volume fraction ratio was 7.74 ± 1.60. Considering that each of these peptides contains 11 amide protons in the backbone (the glutamine amide NH_2_ protons are expected to exchange too slowly to detect^[15]^) and 3 indole NH protons, this ratio is greater than the expected number (3.67), suggesting that the Trp indole NH protons are less water accessible than the backbone amide NH protons or that the Trp indole NH protons in each peptide have non-overlapping chemical shifts. In addition to the amide and Trp indole NH CEST peaks at ~5.4 ppm and ~3.5 ppm, respectively, some of the peptides containing arginine (Arg, R) also showed a peak at ~2 ppm arising from guanidyl proton exchange.

**Table S2.** Proton exchange rates for amine, amide, and Trp NH indole pools obtained from various peptide samples using spectral fitting and QUESP analysis.

| **Peptide** | *Amine NH_3_* | | | | |
| --- | --- | --- | --- | --- | --- |
|  | ***f*_s_** | ***f*_s_ fit error** | ***k*_sw_ (s^-1^)** | ***k*_sw_ fit error (s^-1^)** | ***R*^2^** |
| KMWDWEQKKKWI | 33.4 x 10^-6^ | 15 x 10^-6^ | 2017 | 1136 | 0.995 |
| IWKKKQEWDWMK | 72.8 x 10^-6^ | 130 x 10^-6^ | 5517 | 10336 | 0.999 |
| KMWWEQKKKWI | 33.8 x 10^-6^ | 5.3 x 10^-6^ | 2610 | 483 | 1.000 |
| KMWWQKKKWI | 25.4 x 10^-6^ | 5.5 x 10^-6^ | 1382 | 499 | 0.993 |
| KMWKWEQKKKWI | 21.2 x 10^-6^ | 5.4 x 10^-6^ | 1205 | 628 | 0.978 |
| KMWDWRQKKKWI | 113 x 10^-6^ | 4.2 x 10^-6^ | 1660 | 91 | 1.000 |
| KMWKWRQKKKWI | 138 x 10^-6^ | 7.4 x 10^-6^ | 1981 | 139 | 1.000 |
| KMWNWEQKKKWI | 32.4 x 10^-6^ | 1.6 x 10^-6^ | 1452 | 118 | 1.000 |
| KMWNWQQKKKWI | 31.5 x 10^-6^ | 1.9 x 10^-6^ | 1454 | 143 | 0.999 |
| KMWIWEQKKKWI | 44.8 x 10^-6^ | 11 x 10^-6^ | 2640 | 745 | 0.999 |
| KMWIWLQKKKWI | 31.8 x 10^-6^ | 3.5 x 10^-6^ | 1854 | 279 | 0.999 |
| WDWKKIKQKEMW | 86.5 x 10^-6^ | 420 x 10^-6^ | 6836 | 33719 | 0.996 |
| GGGSWKWRGGGS | 213 x 10^-6^ | 16 x 10^-6^ | 1610 | 184 | 0.999 |
| GGGSWKWRSGGG | 208 x 10^-6^ | 82 x 10^-6^ | 2175 | 1089 | 0.997 |
| WKWRGGGSWKWR | 261 x 10^-6^ | 19 x 10^-6^ | 1538 | 175 | 0.999 |

| **Peptide** | *Amide NH* | | | | |
| --- | --- | --- | --- | --- | --- |
|  | ***f*_s_** | ***f*_s_ fit error** | ***k*_sw_ (s^-1^)** | ***k*_sw_ fit error (s^-1^)** | ***R*^2^** |
| KMWDWEQKKKWI | 36.4 x 10^-6^ | 1.3 x 10^-6^ | 495 | 52 | 0.993 |
| IWKKKQEWDWMK | 36.1 x 10^-6^ | 1.4 x 10^-6^ | 491 | 54 | 0.992 |
| KMWWEQKKKWI | 43.1 x 10^-6^ | 1.5 x 10^-6^ | 531 | 63 | 0.992 |
| KMWWQKKKWI | 41.2 x 10^-6^ | 1.8 x 10^-6^ | 378 | 34 | 0.992 |
| KMWKWEQKKKWI | 62.9 x 10^-6^ | 4.0 x 10^-6^ | 416 | 59 | 0.982 |
| KMWDWRQKKKWI | 49.1 x 10^-6^ | 1.9 x 10^-6^ | 501 | 55 | 0.992 |
| KMWKWRQKKKWI | 96.6 x 10^-6^ | 1.9 x 10^-6^ | 549 | 37 | 0.997 |
| KMWNWEQKKKWI | 57.4 x 10^-6^ | 4.4 x 10^-6^ | 381 | 59 | 0.975 |
| KMWNWQQKKKWI | 56.6 x 10^-6^ | 1.5 x 10^-6^ | 416 | 25 | 0.997 |
| KMWIWEQKKKWI | 44.3 x 10^-6^ | 1.8 x 10^-6^ | 440 | 44 | 0.992 |
| KMWIWLQKKKWI | 47.9 x 10^-6^ | 1.8 x 10^-6^ | 354 | 24 | 0.995 |
| WDWKKIKQKEMW | 36.3 x 10^-6^ | 2.3 x 10^-6^ | 470 | 79 | 0.978 |
| GGGSWKWRGGGS | 155 x 10^-6^ | 2.5 x 10^-6^ | 767 | 52 | 0.999 |
| GGGSWKWRSGGG | 144 x 10^-6^ | 3.0 x 10^-6^ | 946 | 59 | 0.999 |
| WKWRGGGSWKWR | 113 x 10^-6^ | 1.4 x 10^-6^ | 759 | 40 | 0.999 |

| **Peptide** | *Trp indole NH* | | | | |
| --- | --- | --- | --- | --- | --- |
|  | ***f*_s_** | ***f*_s_ fit error** | ***k*_sw_ (s^-1^)** | ***k*_sw_ fit error (s^-1^)** | ***R*^2^** |
| KMWDWEQKKKWI | 5.79 x 10^-6^ | 0.16 x 10^-6^ | 743 | 96 | 0.995 |
| IWKKKQEWDWMK | 5.12 x 10^-6^ | 0.29 x 10^-6^ | 785 | 186 | 0.985 |
| KMWWEQKKKWI | 7.50 x 10^-6^ | 0.30 x 10^-6^ | 902 | 120 | 0.997 |
| KMWWQKKKWI | 6.42 x 10^-6^ | 0.52 x 10^-6^ | 398 | 71 | 0.971 |
| KMWKWEQKKKWI | 6.50 x 10^-6^ | 0.69 x 10^-6^ | 551 | 203 | 0.920 |
| KMWDWRQKKKWI | 5.79 x 10^-6^ | 0.25 x 10^-6^ | 644 | 121 | 0.984 |
| KMWKWRQKKKWI | 9.96 x 10^-6^ | 0.30 x 10^-6^ | 726 | 101 | 0.994 |
| KMWNWEQKKKWI | 5.92 x 10^-6^ | 0.10 x 10^-6^ | 703 | 57 | 0.998 |
| KMWNWQQKKKWI | 5.93 x 10^-6^ | 0.16 x 10^-6^ | 748 | 95 | 0.995 |
| KMWIWEQKKKWI | 6.46 x 10^-6^ | 0.26 x 10^-6^ | 771 | 139 | 0.991 |
| KMWIWLQKKKWI | 6.22 x 10^-6^ | 0.27 x 10^-6^ | 718 | 149 | 0.985 |
| WDWKKIKQKEMW | 6.31 x 10^-6^ | 0.47 x 10^-6^ | 508 | 113 | 0.967 |
| GGGSWKWRGGGS | 14.5 x 10^-6^ | 0.57 x 10^-6^ | 931 | 121 | 0.997 |
| GGGSWKWRSGGG | 13.2 x 10^-6^ | 2.0 x 10^-6^ | 1203 | 394 | 0.990 |
| WKWRGGGSWKWR | 8.51 x 10^-6^ | 0.33 x 10^-6^ | 703 | 85 | 0.994 |


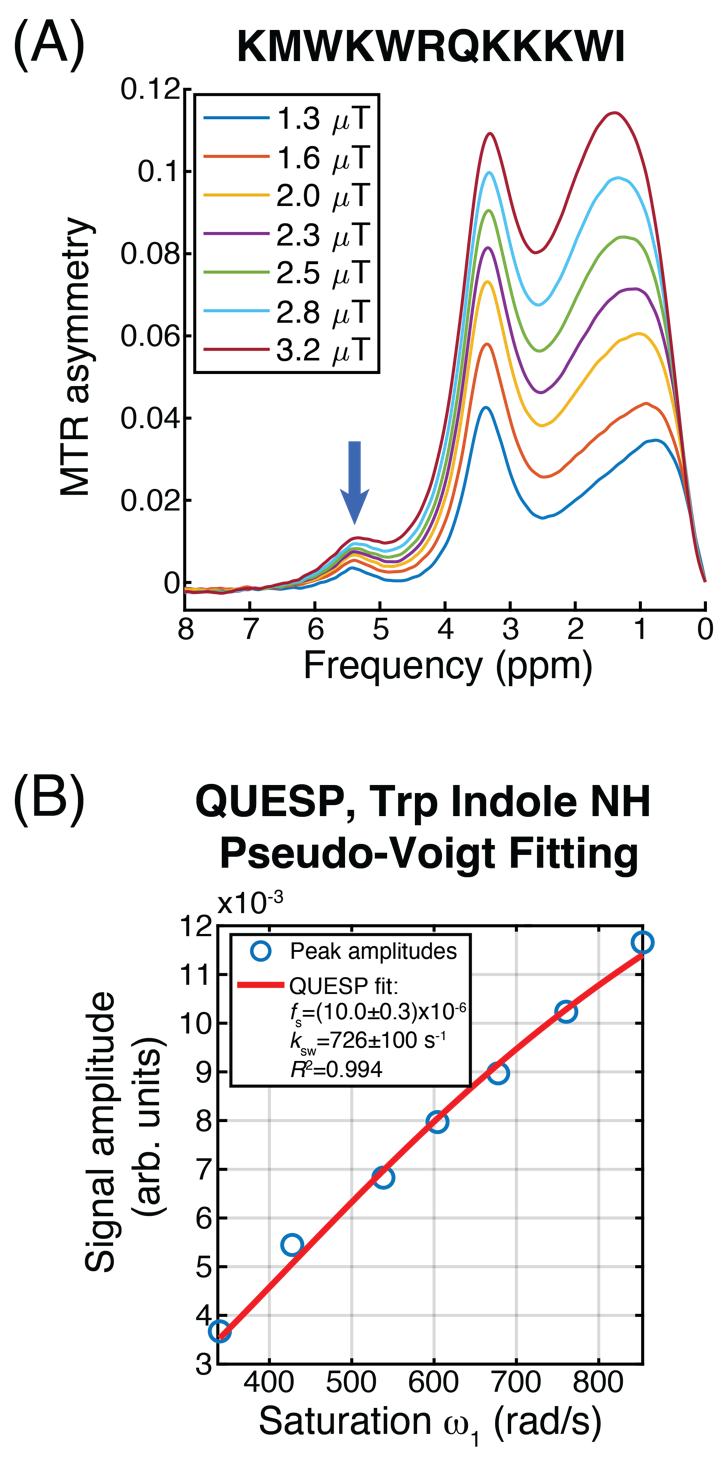


**Figure S2.** Representative data from the investigation into Trp indole NH exchange with ^1^H CEST NMR spectroscopy, obtained from peptide KMWKWRQKKKWI. (A) *MTR*_asym_ profiles for different saturation pulse amplitudes, ω_1_. The blue arrow indicates the peak arising from indole NH proton exchange at 5.4 ppm. (B) QUESP analysis on the fitted indole NH peak amplitudes to determine the proton volume fraction (*f*_s_) and exchange rate (*k*_sw_).

## QUESP analysis of proton pool exchange in peptide KMWDWQKKKWI

**Figure S3** below shows the analysis of the E-removed peptide, which demonstrated the additional CEST proton pool at 4.2 ppm. Representative Pseudo-Voigt fitting is shown for one of the CEST spectra used to extract peak amplitudes for QUESP analysis, as well as the QUESP fitting of the peak amplitudes to determine the proton exchange rates.


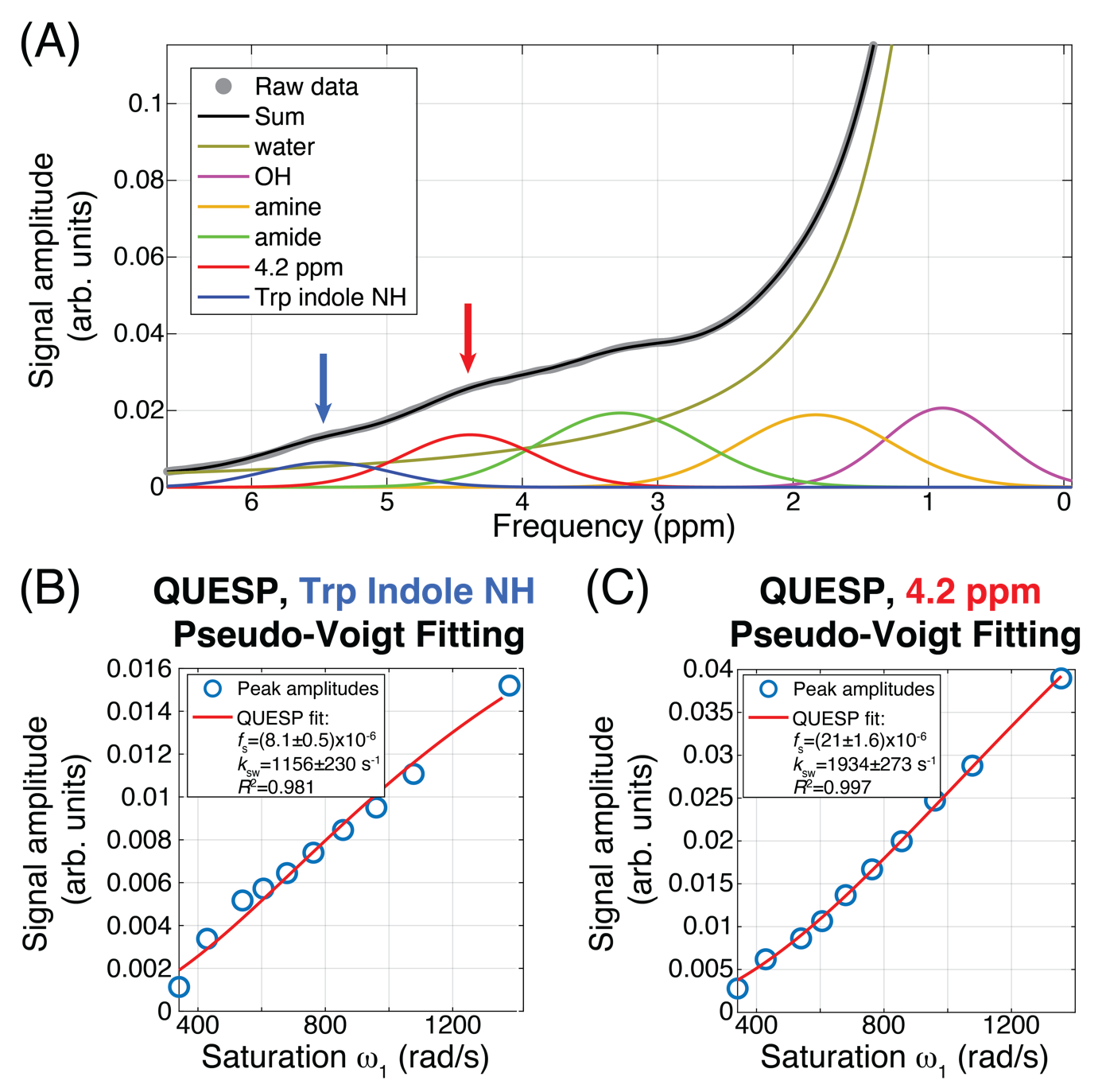


**Figure S3*.*** Spectral and QUESP fitting of the E-removed Trp peptide. (A) Representative CEST pool fitting with Pseudo-Voigt peaks for the CEST spectrum acquired with a saturation *B*_1_ amplitude of 2.54 μT. (B-C) QUESP fitting of the CEST peak amplitudes obtained from spectral fitting, for (B) Trp indole NH; and (C) the 4.2 ppm pool. The peptide concentration was 7.67 mg/mL.

## CEST NMR investigation of other amino acid substitution effects on the 4.2 ppm signal

**Figure S4** shows the MTR asymmetry plots from other peptides derived from the peptide sequence shown in **Figure 2C** of the main text, in order to determine which Lys residue is involved in producing the 4.2 ppm CEST signal. Re-introduction of Lys at positions 7 and 9 in the peptide sequence did not significantly re-introduce the 4.2 ppm signal into the CEST NMR spectra, suggesting that neither the K7 nor the K9 residue is involved in the cation-π interaction causing the 4.2 ppm pool.


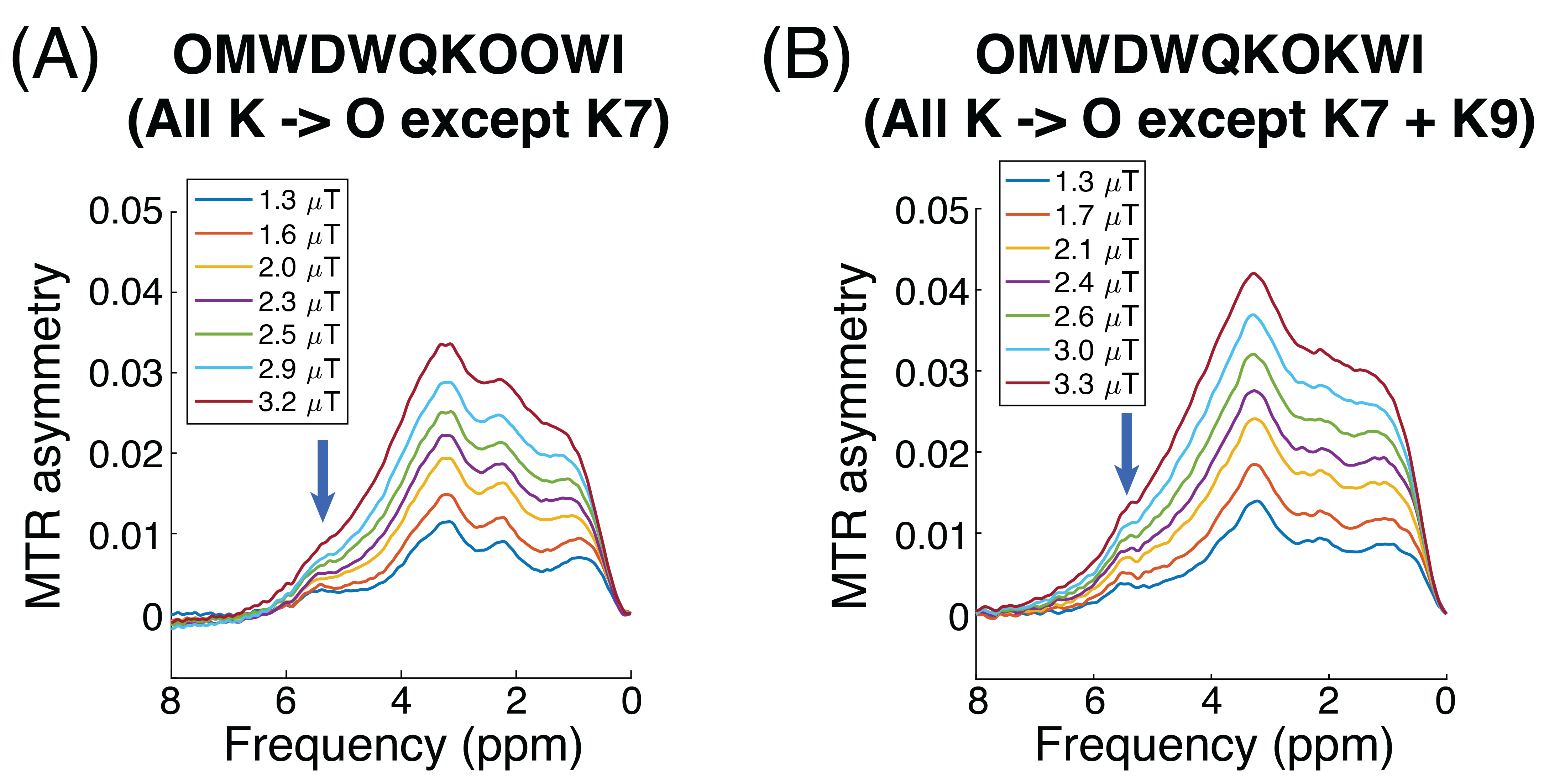


**Figure S4*.*** Additional amino acid substitutions tested on the peptide sequence KMWDWQKKKWI giving rise to the 4.2 ppm peak. (A) Re-introduction of K7 into the O-substituted peptide. (B) Re-introduction of both K7 and K9 into the O-substituted peptide. The peptide concentrations for the (A) and (B) samples were 6.67 mg/mL and 8.00 mg/mL, respectively.

## Peptide structure simulation with PEP-FOLD4

**Figure S5** includes the lowest-energy predicted structures of the base Trp peptide without the 4.2 ppm CEST peak (KMWDWEQKKKWI) and the E-removed peptide in which the 4.2 ppm peak appears (KMWDWQKKKWI). The two lowest-energy conformers of the original peptide appear to demonstrate an interaction between the K1 and E6 side-chains. The “E-removed” peptide suggests an interaction between the K1 and W3 residues for the lowest-energy conformer and possibly for the highest-energy prediction. Importantly, a relatively large energy gap separates the two conformations of lowest energy (~3 kcal/mol) for the E-removed peptide.


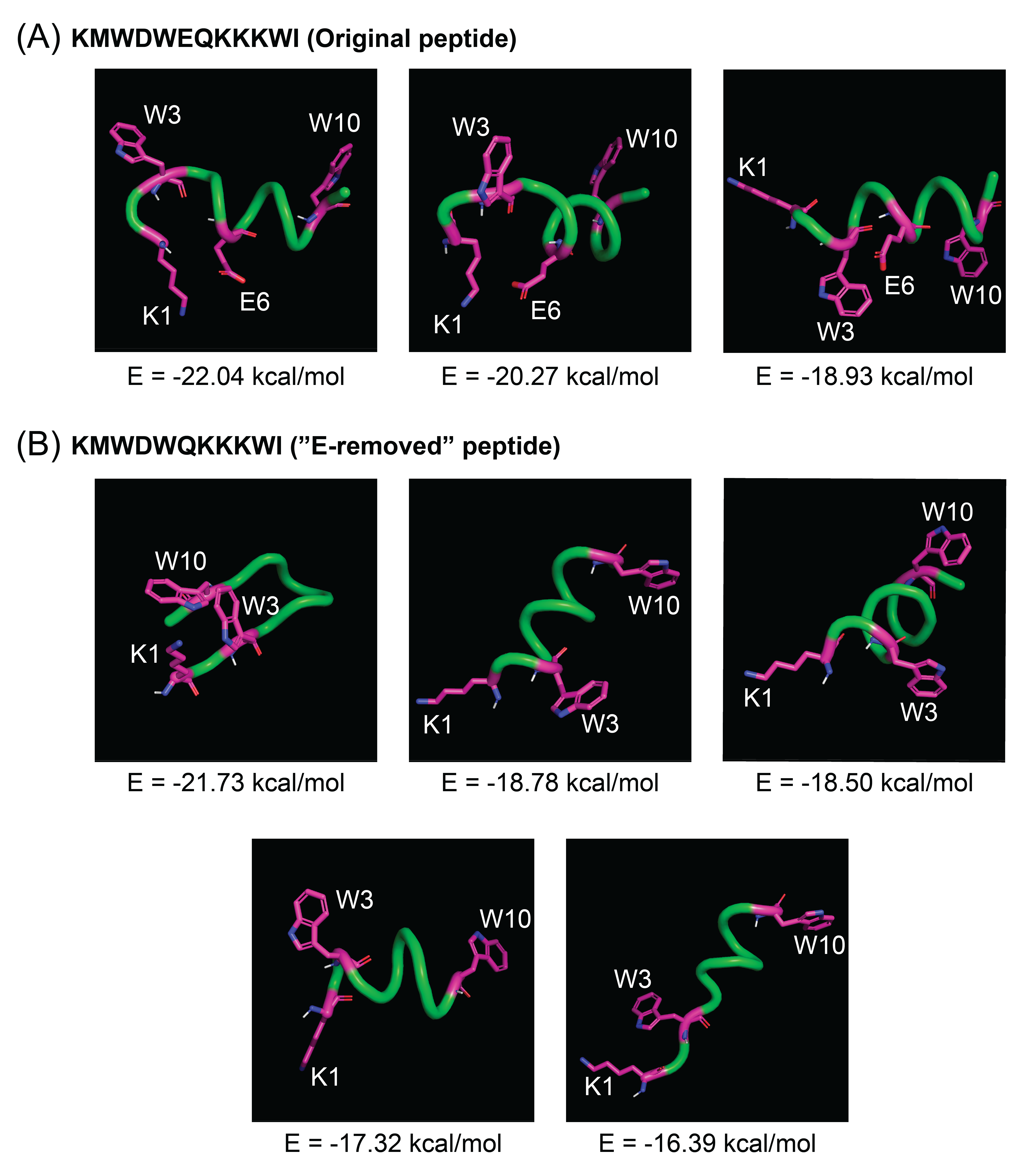


**Figure S5*.*** Peptide structure predictions for (A) the original Trp peptide sequence; and (B) the same sequence with the E6 residue removed, which demonstrates a novel CEST peak at 4.2 ppm. The calculated energy is displayed below each predicted structure and are arranged in increasing energy values from left to right, then top to bottom. The leftmost conformations are shown in the main text, as **Figure 3A-B**.

## CD spectroscopy of short peptides

We performed CD spectroscopy on the peptides investigated regarding the CEST peak at 4.2 ppm. **Figure S6** summarizes the results. We observed that all peptides but two exhibited very similar spectral features, particularly a positive peak at 228 nm and a negative peak at 208 nm. The two peptides with a different spectral profile were KMWDWEQKKKWI and KM[5-HTP]DWQKKKWI, both of which had a slight positive peak at 228 nm and a negative peak at 199 nm. These results suggest that residue W3 plays a prominent role in driving peptide secondary structure when the E6 residue is removed from the original peptide, similar to what we observed in our simulated structures (**Figure S5**).


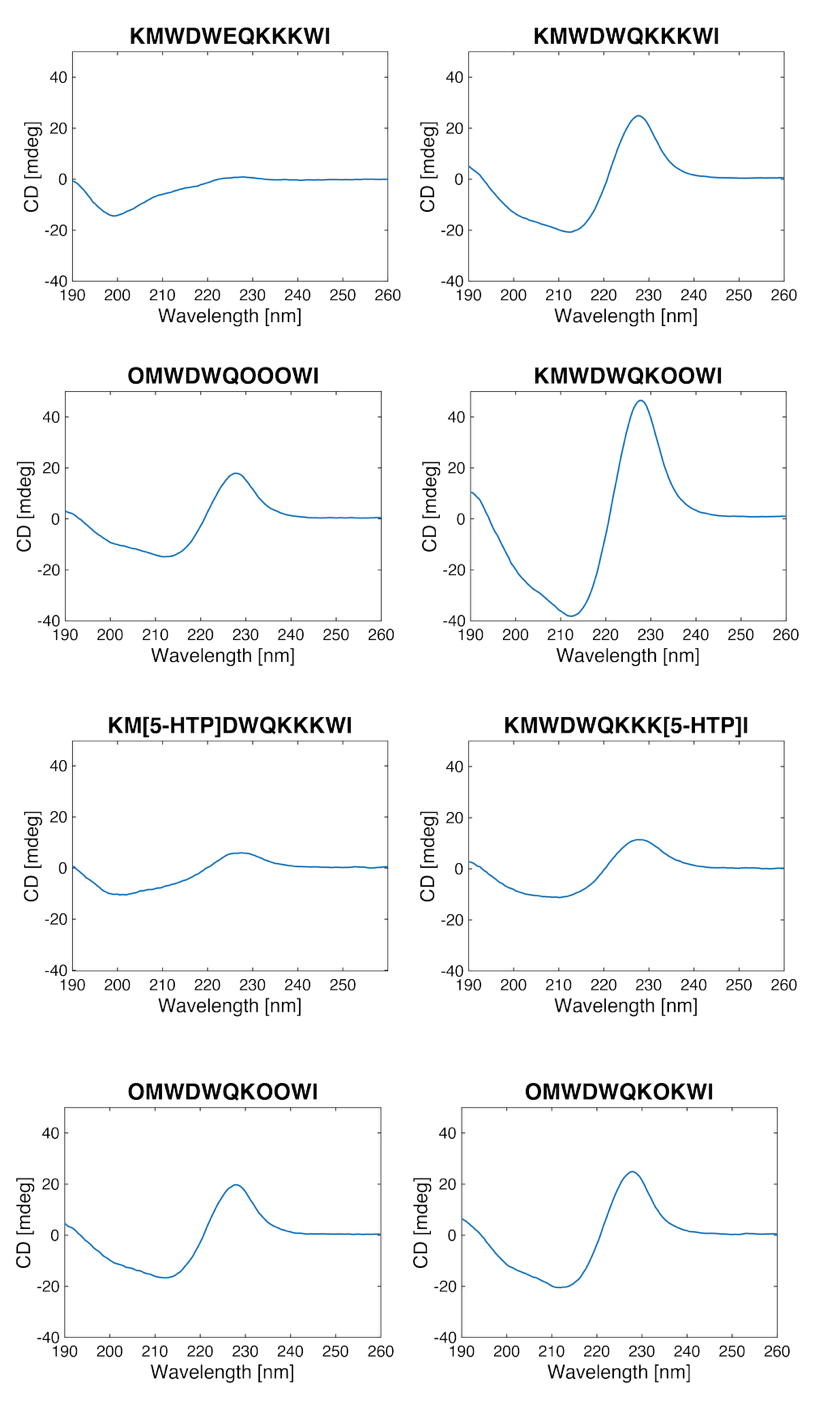


**Figure S6.** CD spectroscopy results of the Trp-containing peptides investigated regarding the origin of the 4.2 ppm CEST peak.

## Fluorescence spectroscopy of short peptides

**Figure S7** shows the fluorescence spectra obtained for the peptides investigated regarding the CEST peak at 4.2 ppm. While all peptides demonstrate a fluorescence emission peak at about 350 nm, only the KMWDWEQKKKWI peptide shows a significant emission peak at about 420 nm. Notably, removal of the E6 residue leads to a dramatic enhancement of the 350 nm peak, suggesting significant structural changes. The quenching of the 420 nm peak observed in the other peptides may be due to an interaction between the W3 and W10 residues, as predicted by structural simulation (**Figure S5**).


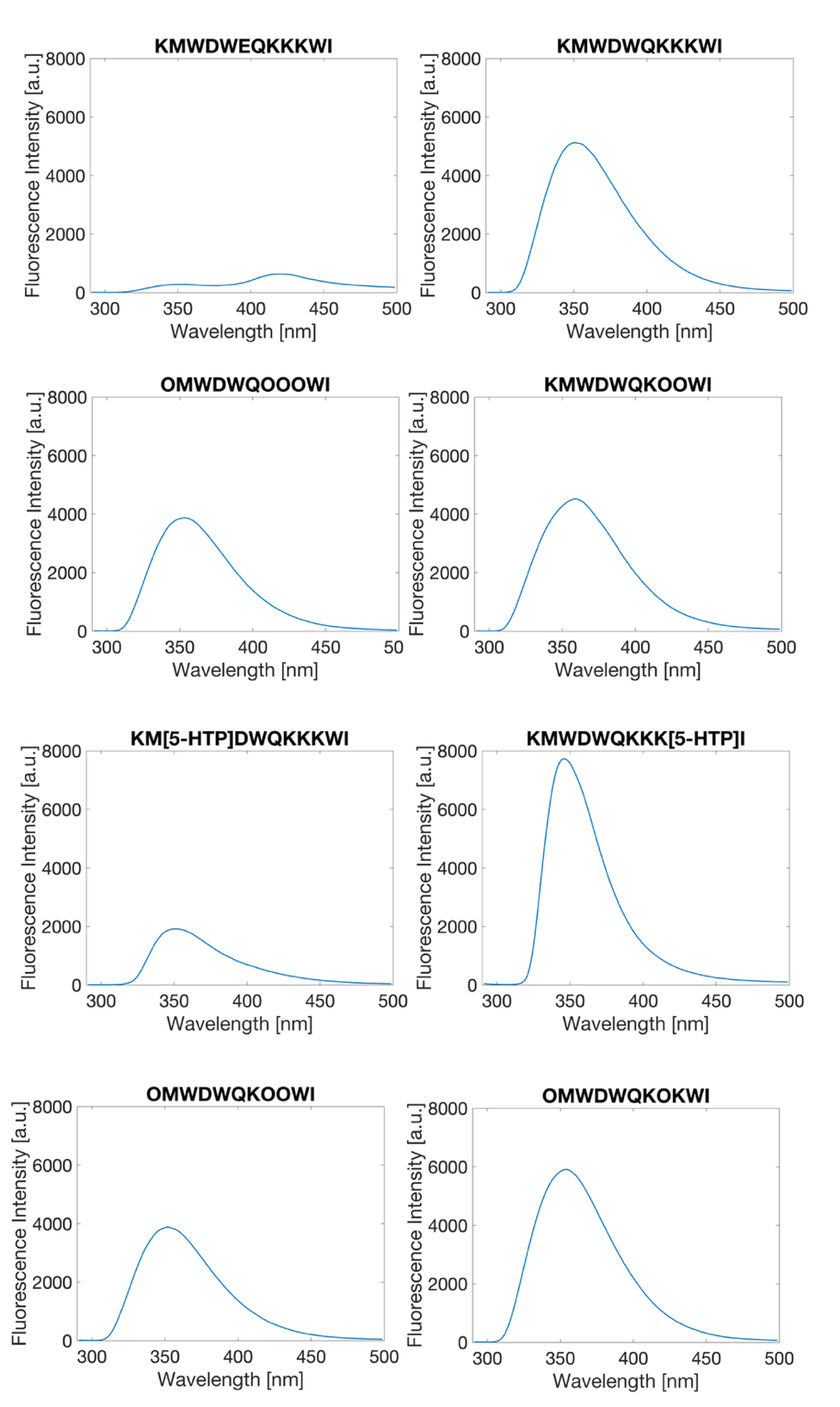


**Figure S7.** Fluorescence spectroscopy of Trp in the investigated peptides.

## Spectral fitting and QUESP analysis of glucoamylase CEST spectra

**Figure S8** demonstrates the difference between glucoamylase CEST spectra acquired at 20 and 37 °C (both pH 7.3), as well as the results of spectral fitting and QUESP analysis of the CEST peaks at 7.3 and 9.8 ppm for the 37 °C dataset. The 9.8 ppm CEST peak appears to broaden out a little bit at 37 °C, and both peaks appear to remain similar in amplitude, although it must be accounted for that the water *T*_1_ is longer at 37 °C but both temperatures use an equal saturation duration, meaning that the 37 °C data are farther from saturation steady-state than the 20 °C data.


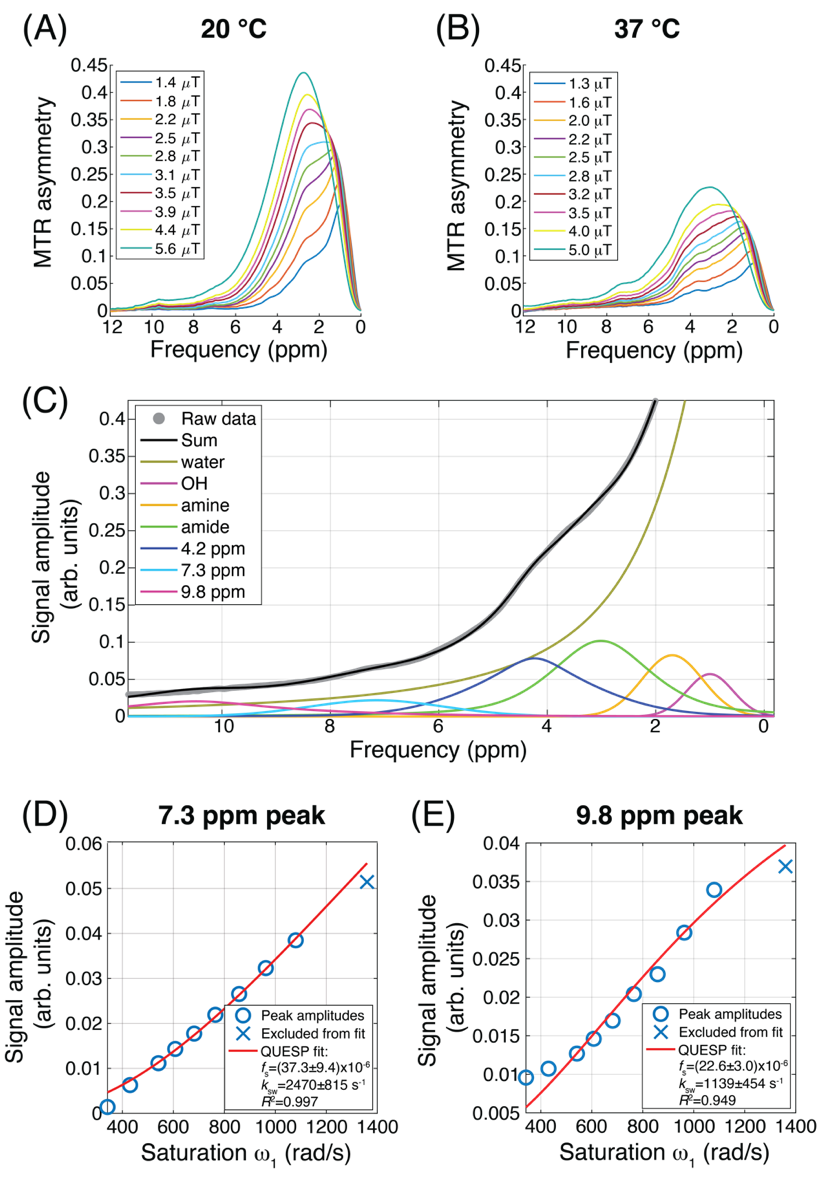

**Figure S8*.*** Temperature dependence study, spectral fitting, and QUESP fitting of glucoamylase. (A-B) Comparison between CEST spectroscopy at (A) 20 °C; and (B) 37 °C (same as **Figure 4A** in the main text). (C-E) Spectral fitting and QUESP analysis of 37 °C data: (C) Representative CEST pool fitting with Pseudo-Voigt peaks for the CEST spectrum acquired with a saturation *B*_1_ amplitude of 2.81 μT. (D-E) QUESP fitting of the fitted CEST peak amplitudes for (D) the 7.3 ppm pool; and (E) the 9.8 ppm pool. Note that the highest saturation *B*_1_ amplitude was excluded for both fits due to poor CEST spectral fitting. The protein concentration was 57.5 mg/mL.

## In vitro CEST and CESL spectroscopic imaging of glucoamylase and poly-L-lysine

**Figure S9** below is an extended figure of **Figure 5** in the main manuscript which displays the results at all measured saturation amplitudes. Whereas the amide contrast at 3.5 ppm plateaus at 5 μT saturation and even decreases slightly at the highest amplitudes, the 7.3 ppm contrast continues to monotonically increase with saturation amplitude, reaching a similar level at 9 μT as the PLL does at 2 μT. The amount of contrast per exchangeable proton at 7.3 ppm increases monotonically as well, reflecting the increasing labeling efficiency of the stronger saturation pulse. **Table S3** reports the voxelwise mean and standard deviation of the MTR asymmetry values for each tube.


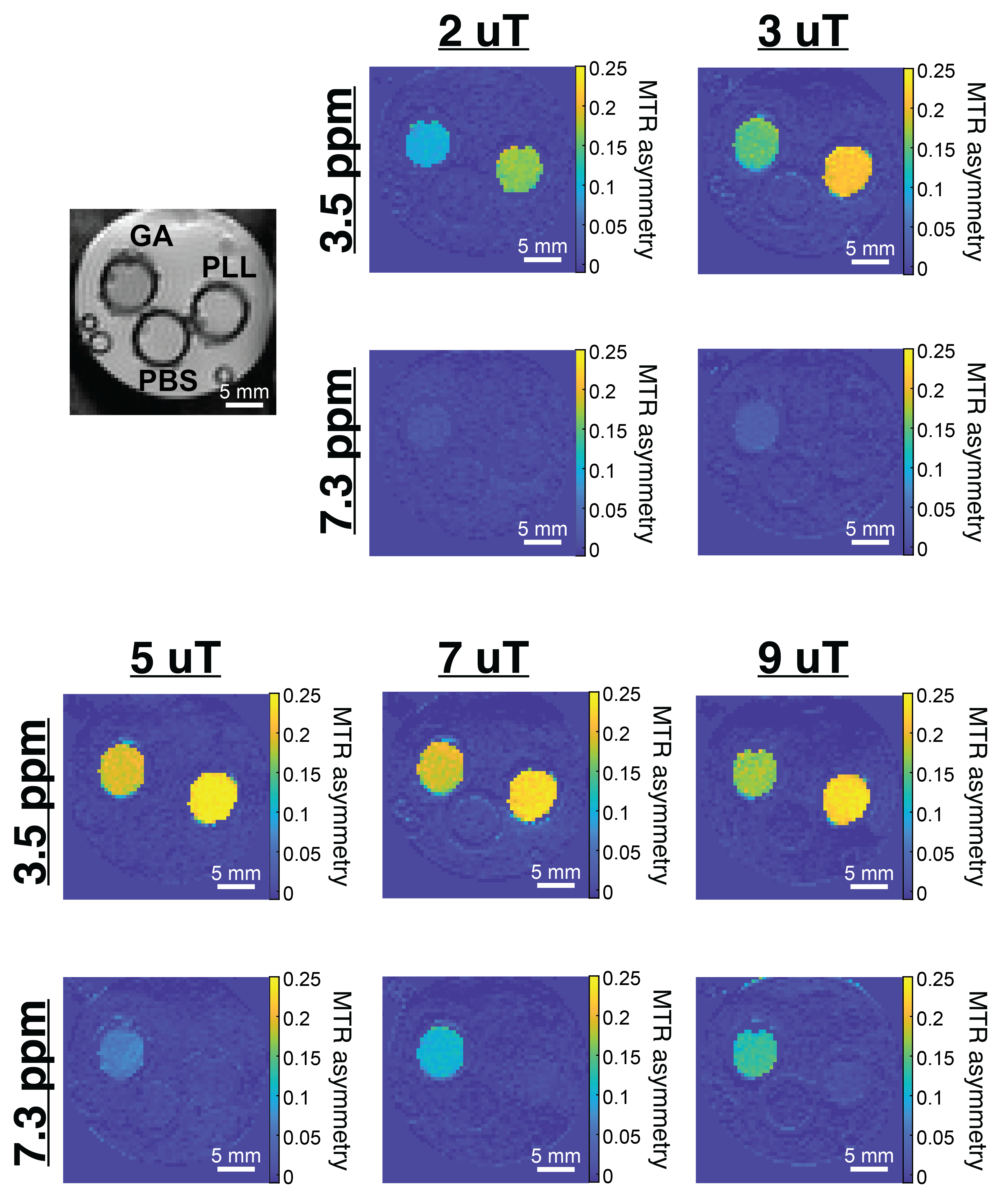


**Figure S9.** Extended version of **Figure 5** in the main manuscript showing additional *MTR*_asym_ images for all measured saturation amplitudes.

**Table S3.** ROI statistics of MTR asymmetry values *vs.* saturation amplitude for protein imaging sample tubes shown in **Figure S9**. Values are reported as mean ± standard deviation.

| **Sample** | **Saturation amplitude (μT)** | ***MTR*_asym_(3.5ppm)** | ***MTR*_asym_(7.3ppm)** |
| --- | --- | --- | --- |
| Poly-L-lysine | 2 | 0.1656 ± 0.0054 | 0.0022 ± 0.0041 |
|  | 3 | 0.2112 ± 0.0060 | -0.0004 ± 0.0044 |
|  | 5 | 0.2389 ± 0.0050 | 0.0036 ± 0.0039 |
|  | 7 | 0.2267 ± 0.0068 | 0.0041 ± 0.0047 |
|  | 9 | 0.2305 ± 0.0066 | 0.0086 ± 0.0050 |
| Glucoamylase | 2 | 0.0979 ± 0.0065 | 0.0136 ± 0.0053 |
|  | 3 | 0.1487 ± 0.0066 | 0.0275 ± 0.0043 |
|  | 5 | 0.1918 ± 0.0061 | 0.0633 ± 0.0054 |
|  | 7 | 0.1885 ± 0.0079 | 0.1053 ± 0.0058 |
|  | 9 | 0.1698 ± 0.0082 | 0.1318 ± 0.0090 |
| PBS | 2 | 0.0008 ± 0.0044 | 0.0028 ± 0.0049 |
|  | 3 | 0.0044 ± 0.0052 | 0.0002 ± 0.0042 |
|  | 5 | -0.0008 ± 0.0056 | 0.0040 ± 0.0043 |
|  | 7 | 0.0056 ± 0.0063 | -0.0038 ± 0.0047 |
|  | 9 | -0.0057 ± 0.0053 | -0.0031 ± 0.0056 |

**Figure S10** shows the CESL contrast within each tube as a function of the spin-lock pulse amplitude and duration. The contrast varies little with the locking amplitude, as predicted by theory,^[16]^ whereas the overall signal decreases as the locking time increases, except for the shortest locking time (50 ms), which may be due to hardware limitations during the balanced spin-lock. The glucoamylase tube signal decreases faster than the signal in the PLL or PBS tubes, creating strong glucoamylase contrast.


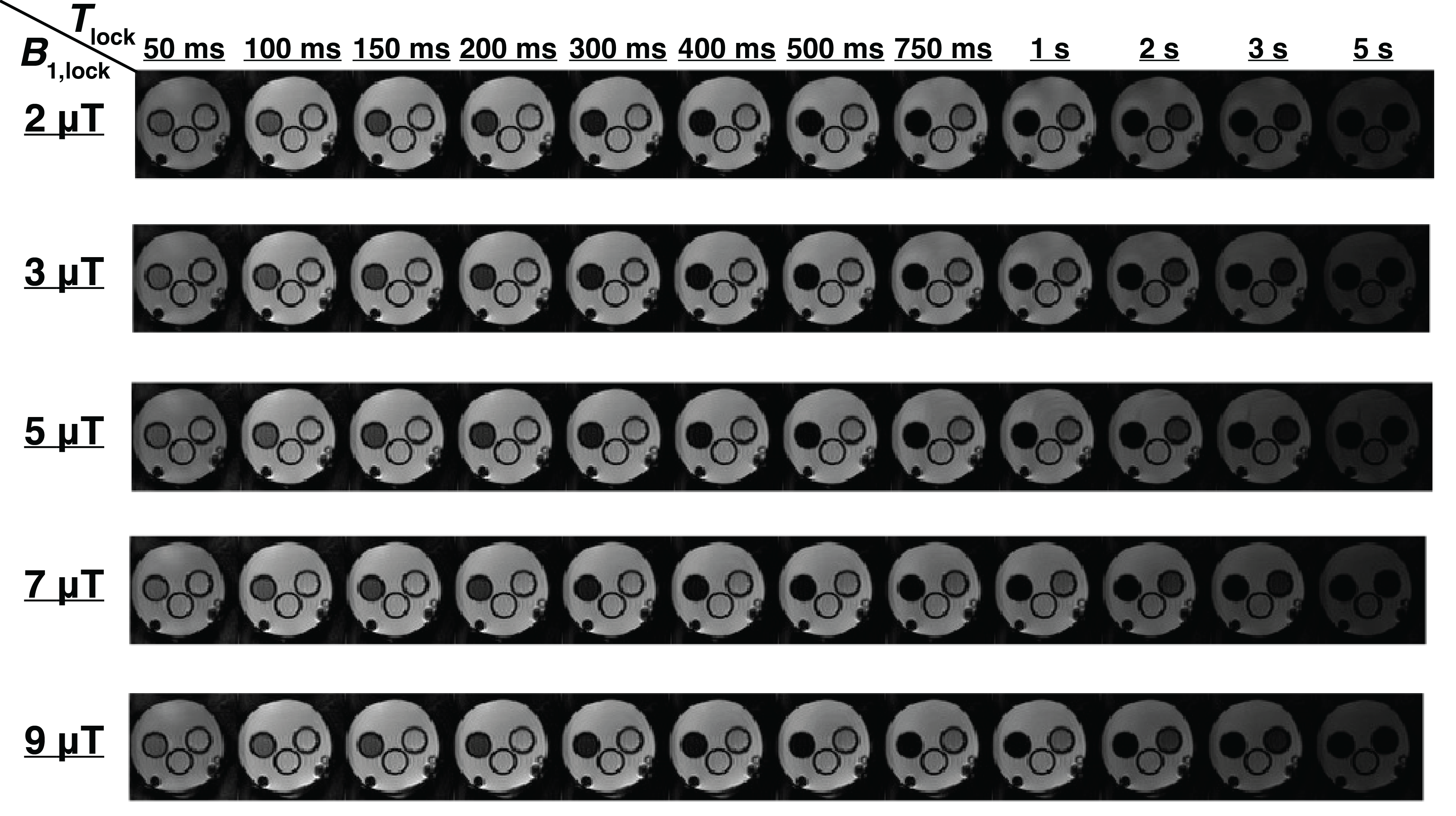


**Figure S10.** Chemical exchange spin-locking contrast between glucoamylase, poly-L-lysine, and phosphate-buffered saline as a function of spin-lock amplitude and duration.

## In vivo CEST spectroscopic imaging in mouse brain

**Figure S11** shows a CEST spectrum out to ±9 ppm in a mouse brain. The main contributions that are discernible are the broad semisolid macromolecular MT pool, which produces the curved baseline, the NOE contribution centered at about -3 ppm, and the amide pool signal at 3.5 ppm. The NOE and amide pools are clearly discernible at 0.7 μT but broaden out at the higher saturation power. These *in vivo* results demonstrate that there are few signals at high ppm values where reporter gene products would be designed to produce CEST signals.


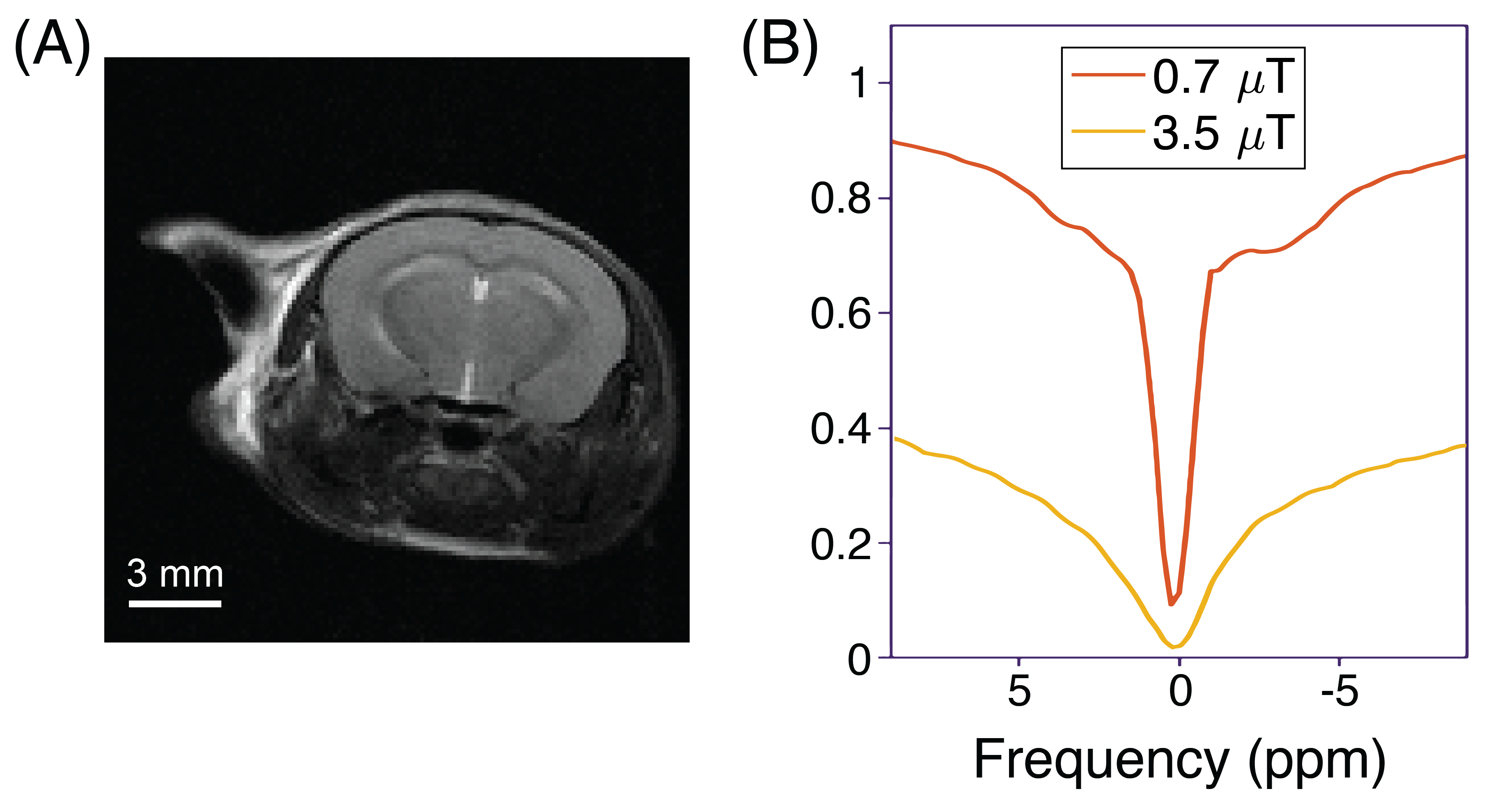


**Figure S11.** *In vivo* CEST imaging results in a mouse brain. (A) Anatomical mouse brain MR image corresponding with the slice used to perform CEST EPI z-spectroscopy. (B) CEST z-spectra averaged over voxels from the whole brain demonstrating a broad baseline due to MT, a significant NOE peak at -3 ppm, and an amide proton pool signal at 3.5 ppm.

# Peptide Characterization

## KMWDWEQKKKWI, HPLC

Sample Name :KMWDWEQKKKWI

Sample ID :U2916HSSG0-1

Time Processed :15:45:29

Month-Day-Year Processed :07/09/2023

Pump A : 0.065% trifluoroacetic in 100% water (v/v)

Pump B : 0.05% trifluoroacetic in 100% acetonitrile (v/v)

Total Flow:1 ml/min

Wavelength:220 nm

<<LC Time Program>>

| Time | Module | Command | Value |
| --- | --- | --- | --- |
| 0.01 | Pumps | B.Conc | 5 |
| 25.00 | Pumps | B.Conc | 65 |
| 25.01 | Pumps | B.Conc | 95 |
| 27.00 | Pumps | B.Conc | 95 |
| 27.01 | Pumps | B.Conc | 5 |
| 35.00 | Pumps | B.Conc | 5 |
| 35.01 | Controller | Stop |  |

<<Column Performance>>

<Detector A>

Column :Inertsil ODS-SP 4.6 x 250 mm

Equipment: GR11010440

<Chromatogram>

mV

min

0

5

10

15

20

25

0

500

1000

1500

2000

Detector A Channel 1 220nm

1

1 / 12.467

2 / 12.798

3 / 13.013

4 / 16.897

<Peak Table>

Detector A Channel 1 220nm

| Peak# | Ret. Time | Area | Height | Area% |
| --- | --- | --- | --- | --- |
| 1 | 12.467 | 343480 | 38342 | 1.356 |
| 2 | 12.798 | 24423820 | 2204861 | 96.424 |
| 3 | 13.013 | 42011 | 6253 | 0.166 |
| 4 | 16.897 | 520187 | 68412 | 2.054 |
| Total |  | 25329498 | 2317868 | 100.000 |

## KMWDWEQKKKWI, Mass Spectrometry


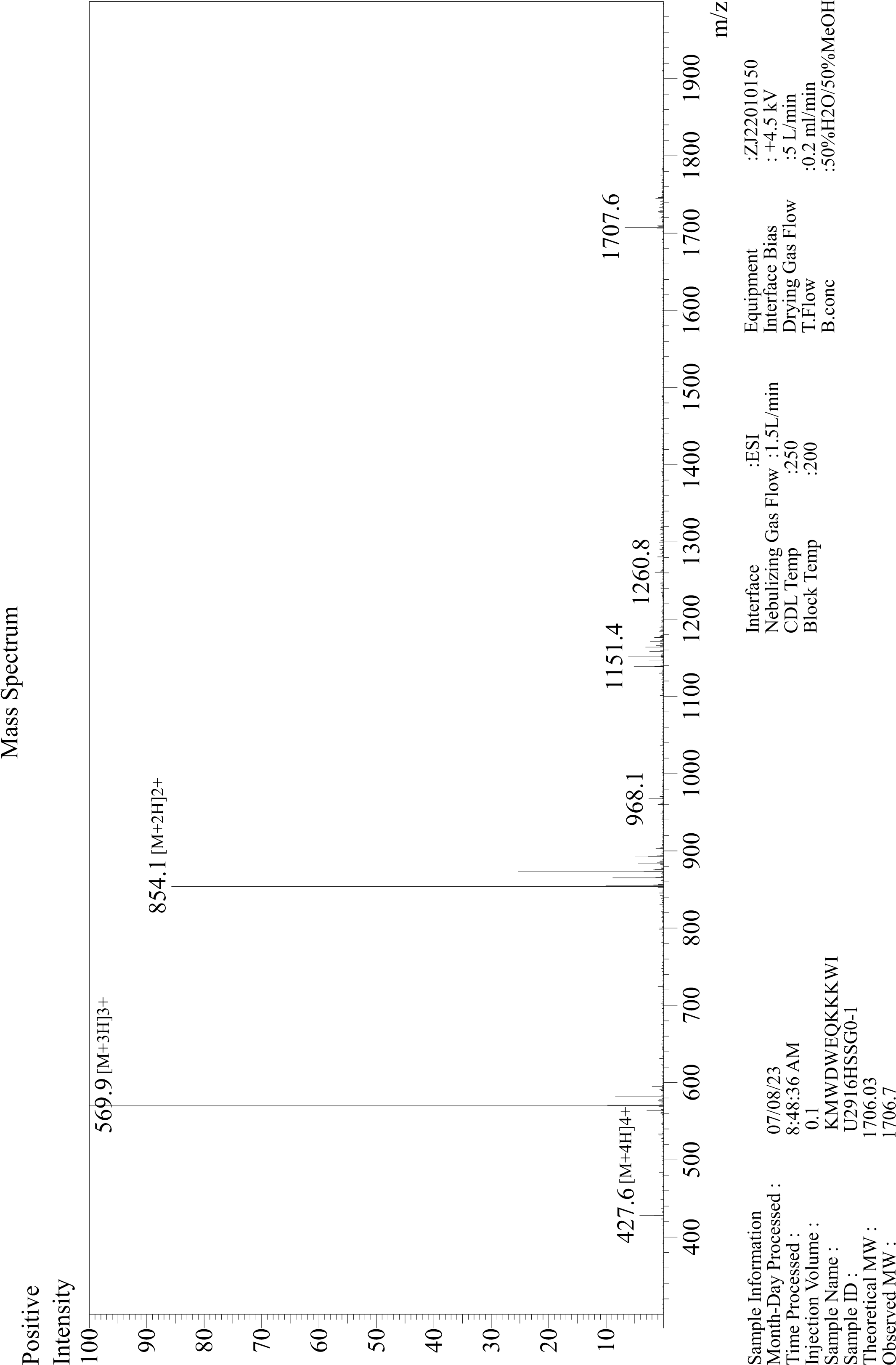


## KMWDWQKKKWI, HPLC

Sample Name :KMWDWQKKKWI

Sample ID :U2916HSSG0-7

Time Processed :7:08:46

Month-Day-Year Processed :07/14/2023

Pump A : 0.065% trifluoroacetic in 100% water (v/v)

Pump B : 0.05% trifluoroacetic in 100% acetonitrile (v/v)

Total Flow:1 ml/min

Wavelength:220 nm

<<LC Time Program>>

| Time | Module | Command | Value |
| --- | --- | --- | --- |
| 0.01 | Pumps | B.Conc | 5 |
| 25.00 | Pumps | B.Conc | 65 |
| 25.01 | Pumps | B.Conc | 95 |
| 27.00 | Pumps | B.Conc | 95 |
| 27.01 | Pumps | B.Conc | 5 |
| 35.00 | Pumps | B.Conc | 5 |
| 35.01 | Controller | Stop |  |

<<Column Performance>>

<Detector A>

Column :Inertsil ODS-SP 4.6 x 250 mm

Equipment: ZJ19010014

<Chromatogram>

mV

min

0

5

10

15

20

25

0

500

1000

1500

Detector A Channel 1 220nm

1

1 / 9.900

2 / 11.125

3 / 11.742

4 / 12.100

5 / 12.392

6 / 12.733

7 / 22.408

<Peak Table>

Detector A Channel 1 220nm

| Peak# | Ret. Time | Area | Height | Area% |
| --- | --- | --- | --- | --- |
| 1 | 9.900 | 8571 | 1085 | 0.036 |
| 2 | 11.125 | 12765 | 1125 | 0.054 |
| 3 | 11.742 | 89928 | 9030 | 0.382 |
| 4 | 12.100 | 22940106 | 1706372 | 97.500 |
| 5 | 12.392 | 224187 | 46005 | 0.953 |
| 6 | 12.733 | 229599 | 23297 | 0.976 |
| 7 | 22.408 | 23197 | 1780 | 0.099 |
| Total |  | 23528353 | 1788694 | 100.000 |

##
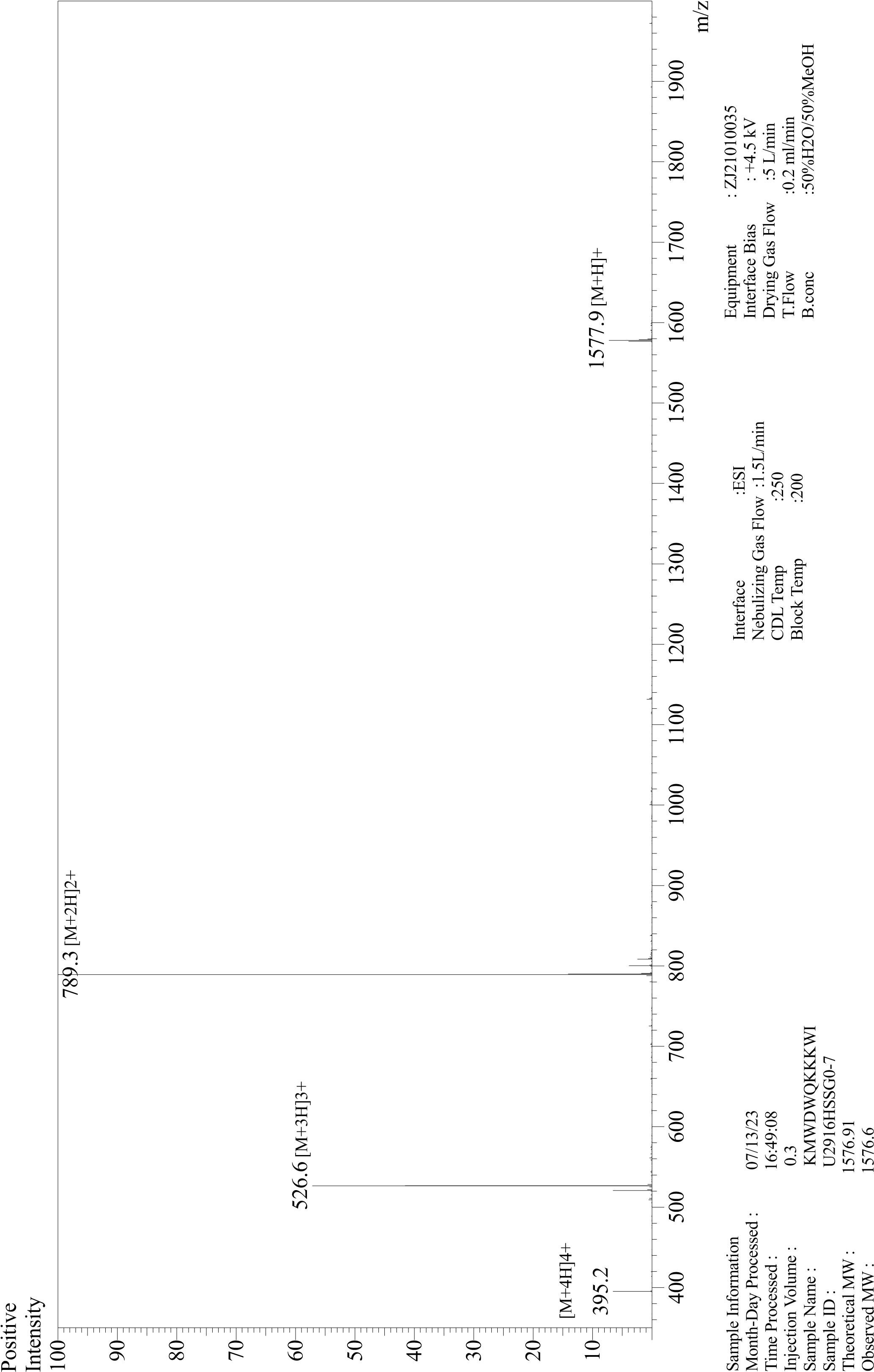
KMWDWQKKKWI, Mass Spectrometry

Mass Spectrum

## OMWDWQOOOWI, HPLC

Sample Name :OMWDWQOOOWI

Sample ID :U3862417G0-11

Time Processed :4:56:07

Month-Day-Year Processed :10/28/2023

Pump A : 0.065% trifluoroacetic in 100% water (v/v)

Pump B : 0.05% trifluoroacetic in 100% acetonitrile (v/v)

Total Flow:1 ml/min

Wavelength:220 nm

<<LC Time Program>>

| Time | Module | Command | Value |
| --- | --- | --- | --- |
| 0.01 | Pumps | Solvent B Conc. | 5 |
| 25.00 | Pumps | Solvent B Conc. | 65 |
| 25.01 | Pumps | Solvent B Conc. | 95 |
| 27.00 | Pumps | Solvent B Conc. | 95 |
| 27.01 | Pumps | Solvent B Conc. | 5 |
| 33.00 | Pumps | Solvent B Conc. | 5 |
| 33.01 | Controller | Stop |  |

<<Column Performance>>

<Detector A>

Column :Inertsil ODS-SP 4.6 x 250 mm

Equipment: ZJ19010015

<Chromatogram>

mV

min

0

5

10

15

20

25

0

500

1000

1500

Detector A Channel 1 220nm

1

1 / 10.767

2 / 10.875

3 / 11.267

4 / 11.467

5 / 11.592

6 / 11.692

7 / 11.842

8 / 17.517

<Peak Table>

Detector A Channel 1 220nm

| Peak# | Ret. Time | Area | Height | Area% |
| --- | --- | --- | --- | --- |
| 1 | 10.767 | 25653 | 4279 | 0.248 |
| 2 | 10.875 | 33077 | 4842 | 0.320 |
| 3 | 11.267 | 10016049 | 1460224 | 96.997 |
| 4 | 11.467 | 53436 | 13286 | 0.517 |
| 5 | 11.592 | 102957 | 13900 | 0.997 |
| 6 | 11.692 | 32290 | 7228 | 0.313 |
| 7 | 11.842 | 40500 | 5312 | 0.392 |
| 8 | 17.517 | 22175 | 3042 | 0.215 |
| Total |  | 10326137 | 1512115 | 100.000 |

##
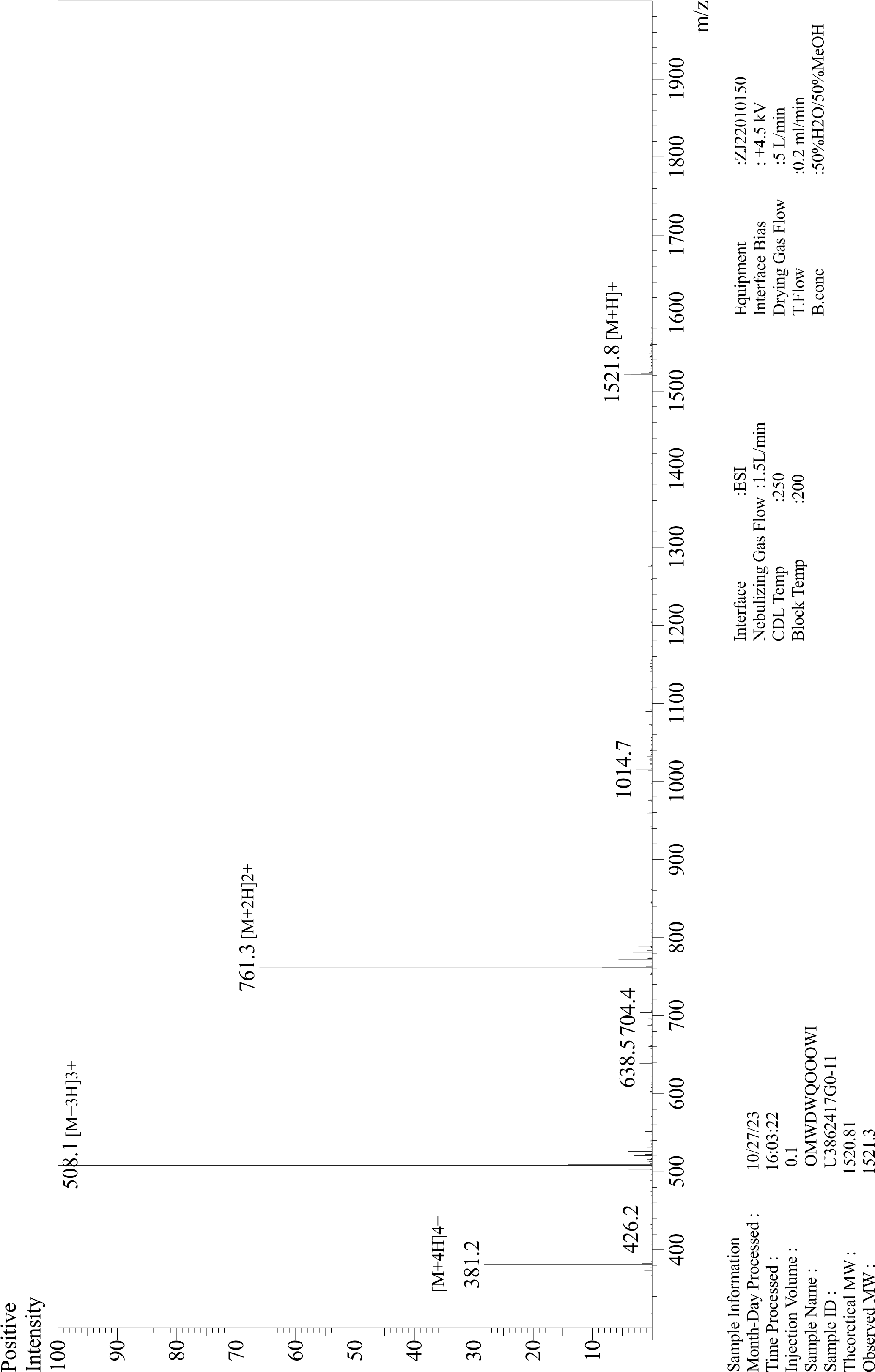
OMWDWQOOOWI, Mass Spectrometry

Mass Spectrum

## KMWDWQKOOWI, HPLC

Sample Name :KMWDWQKOOWI

Sample ID :U534B030G0-1

Time Processed :14:40:04

Month-Day-Year Processed :04/19/2024

Pump A : 0.065% trifluoroacetic in 100% water (v/v)

Pump B : 0.05% trifluoroacetic in 100% acetonitrile (v/v)

Total Flow:1 ml/min

Wavelength:220 nm

<<LC Time Program>>

| Time | Module | Command | Value |
| --- | --- | --- | --- |
| 0.01 | Pumps | B.Conc | 5 |
| 25.00 | Pumps | B.Conc | 65 |
| 25.01 | Pumps | B.Conc | 95 |
| 27.00 | Pumps | B.Conc | 95 |
| 27.01 | Pumps | B.Conc | 5 |
| 35.00 | Pumps | B.Conc | 5 |
| 35.01 | Controller | Stop |  |

<<Column Performance>>

<Detector A>

Column :Inertsil ODS-SP 4.6 x 250 mm

Equipment: GR11010440

<Chromatogram>

mV

min

0

5

10

15

20

25

0

500

1000

1500

2000

Detector A Channel 1 220nm

1

1 / 9.929

2 / 15.645

3 / 15.938

4 / 16.088

<Peak Table>

Detector A Channel 1 220nm

| Peak# | Ret. Time | Area | Height | Area% |
| --- | --- | --- | --- | --- |
| 1 | 9.929 | 114515 | 14917 | 0.726 |
| 2 | 15.645 | 331205 | 47990 | 2.101 |
| 3 | 15.938 | 15290163 | 2337076 | 96.982 |
| 4 | 16.088 | 30132 | 22518 | 0.191 |
| Total |  | 15766015 | 2422501 | 100.000 |

##
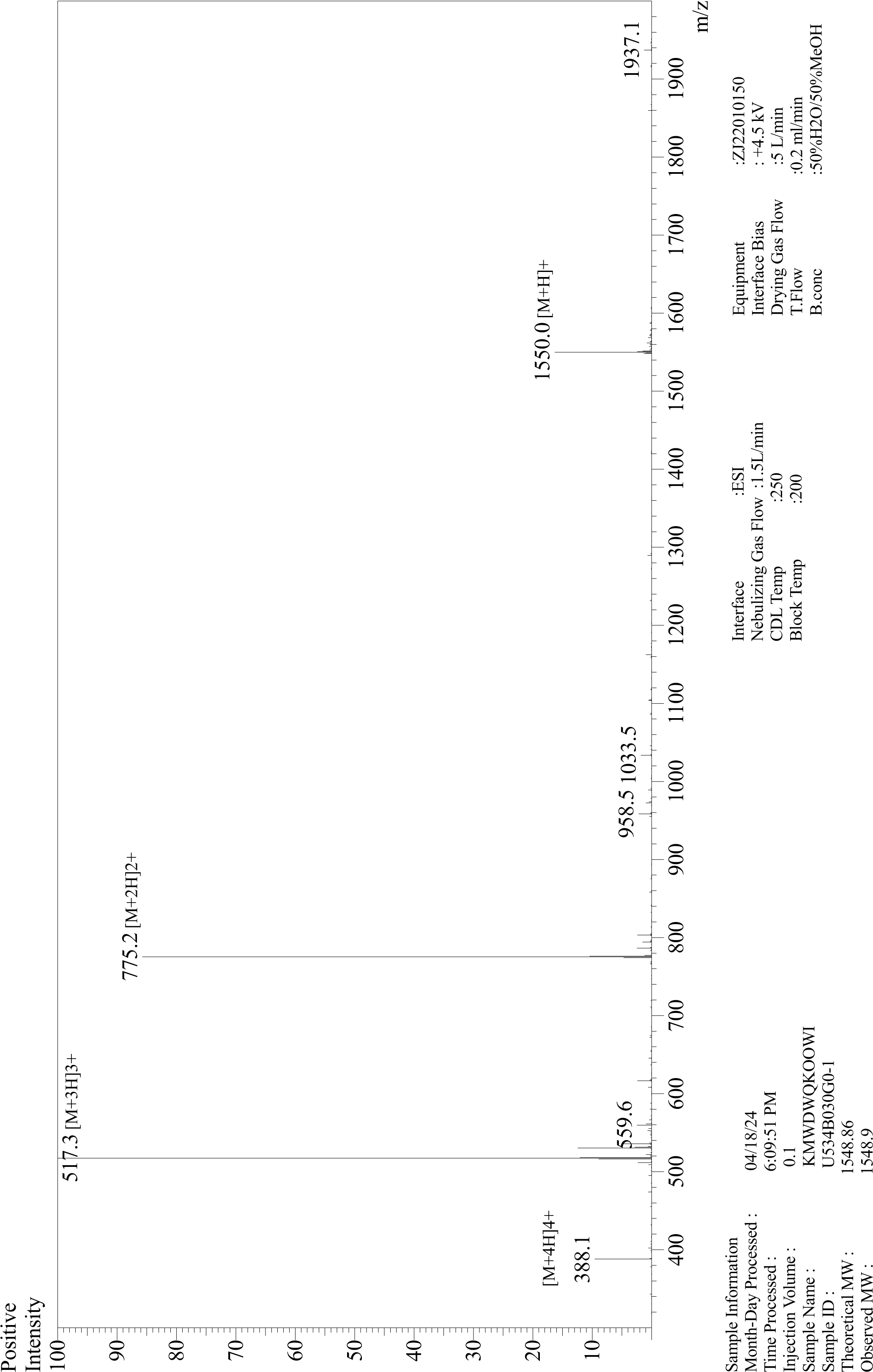
KMWDWQKOOWI, Mass Spectrometry

Mass Spectrum

## KM[5-HTP]DWQKKKWI, HPLC

Sample Name :KM(5HTP)DWQKKKWI

Sample ID :U0751035G0-5

Time Processed :6:41:17

Month-Day-Year Processed :12/03/2023

Pump A : 0.065% trifluoroacetic in 100% water (v/v)

Pump B : 0.05% trifluoroacetic in 100% acetonitrile (v/v)

Total Flow:1 ml/min

Wavelength:220 nm

<<LC Time Program>>

| Time | Module | Command | Value |
| --- | --- | --- | --- |
| 0.01 | Pumps | B.Conc | 5 |
| 25.00 | Pumps | B.Conc | 65 |
| 25.01 | Pumps | B.Conc | 95 |
| 27.00 | Pumps | B.Conc | 95 |
| 27.01 | Pumps | B.Conc | 5 |
| 35.00 | Pumps | B.Conc | 5 |
| 35.01 | Controller | Stop |  |

<<Column Performance>>

<Detector A>

Column :Inertsil ODS-SP 4.6 x 250 mm

Equipment: GK12010012

<Chromatogram>

mV

min

0

5

10

15

20

25

0

250

500

750

1000

Detector A Channel 1 220nm

1

1 / 9.758

2 / 10.525

3 / 12.633

4 / 12.942

5 / 13.242

<Peak Table>

Detector A Channel 1 220nm

| Peak# | Ret. Time | Area | Height | Area% |
| --- | --- | --- | --- | --- |
| 1 | 9.758 | 99807 | 11177 | 1.419 |
| 2 | 10.525 | 64215 | 8516 | 0.913 |
| 3 | 12.633 | 6751502 | 976914 | 96.001 |
| 4 | 12.942 | 73666 | 9260 | 1.047 |
| 5 | 13.242 | 43546 | 5244 | 0.619 |
| Total |  | 7032737 | 1011112 | 100.000 |

## KM[5-HTP]DWQKKKWI, Mass Spectrometry


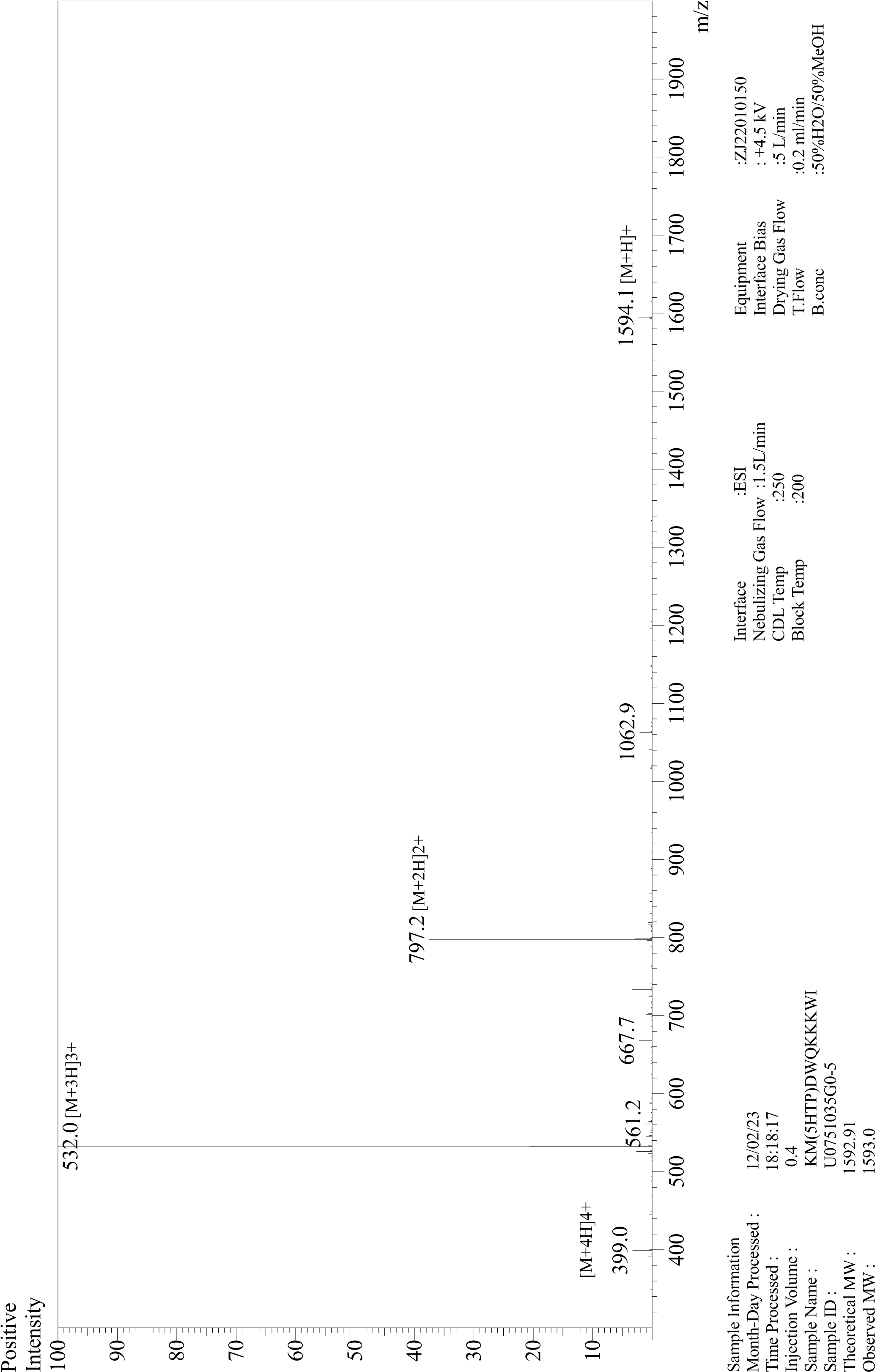


Mass Spectrum

## KMWDWQKKK[5-HTP]I, HPLC

Sample Name :KMWDWQKKK[5-HTP]I

Sample ID :U534B030G0-3

Time Processed :8:30:55

Month-Day-Year Processed :04/21/2024

Pump A : 0.065% trifluoroacetic in 100% water (v/v)

Pump B : 0.05% trifluoroacetic in 100% acetonitrile (v/v)

Total Flow:1 ml/min

Wavelength:220 nm

<<LC Time Program>>

| Time | Module | Command | Value |
| --- | --- | --- | --- |
| 0.01 | Pumps | B.Conc | 5 |
| 25.00 | Pumps | B.Conc | 65 |
| 25.01 | Pumps | B.Conc | 95 |
| 27.00 | Pumps | B.Conc | 95 |
| 27.01 | Pumps | B.Conc | 5 |
| 35.00 | Pumps | B.Conc | 5 |
| 35.01 | Controller | Stop |  |

<<Column Performance>>

<Detector A>

Column :Inertsil ODS-SP 4.6 x 250 mm

Equipment: SS-CM-0309

<Chromatogram>

mV

min

0

5

10

15

20

25

0

500

1000

1500

2000

Detector A Channel 1 220nm

1

1 / 11.085

2 / 11.292

3 / 11.643

4 / 12.116

5 / 12.381

6 / 12.609

7 / 13.043

8 / 19.795

9 / 20.490

<Peak Table>

Detector A Channel 1 220nm

| Peak# | Ret. Time | Area | Height | Area% |
| --- | --- | --- | --- | --- |
| 1 | 11.085 | 11550 | 997 | 0.065 |
| 2 | 11.292 | 9353 | 981 | 0.053 |
| 3 | 11.643 | 55624 | 6726 | 0.315 |
| 4 | 12.116 | 16801286 | 2001748 | 95.076 |
| 5 | 12.381 | 315247 | 45184 | 1.784 |
| 6 | 12.609 | 235768 | 20766 | 1.334 |
| 7 | 13.043 | 18333 | 1551 | 0.104 |
| 8 | 19.795 | 120871 | 8357 | 0.684 |
| 9 | 20.490 | 103397 | 7982 | 0.585 |
| Total |  | 17671430 | 2094290 | 100.000 |

## KMWDWQKKK[5-HTP]I, Mass Spectrometry


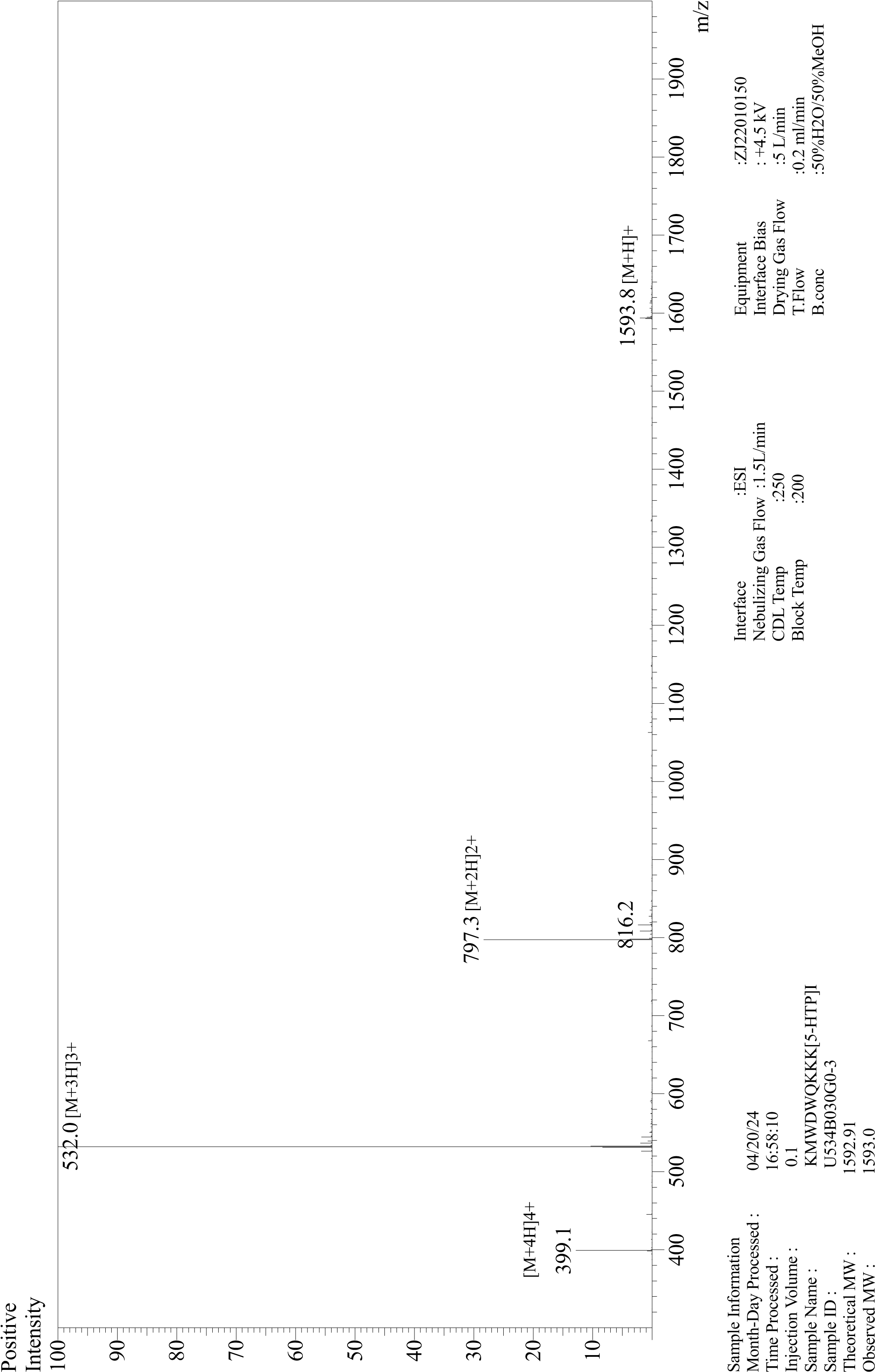


Mass Spectrum

## OMWDWQKOOWI, HPLC

Sample Name :OMWDWQKOOWI

Sample ID :U0751035G0-1

Time Processed :21:32:54

Month-Day-Year Processed :11/21/2023

Pump A : 0.065% trifluoroacetic in 100% water (v/v)

Pump B : 0.05% trifluoroacetic in 100% acetonitrile (v/v)

Total Flow:1 ml/min

Wavelength:220 nm

<<LC Time Program>>

| Time | Module | Command | Value |
| --- | --- | --- | --- |
| 0.01 | Pumps | B.Conc | 5 |
| 25.00 | Pumps | B.Conc | 65 |
| 25.01 | Pumps | B.Conc | 95 |
| 27.00 | Pumps | B.Conc | 95 |
| 27.01 | Pumps | B.Conc | 5 |
| 35.00 | Pumps | B.Conc | 5 |
| 35.01 | Controller | Stop |  |

<<Column Performance>>

<Detector A>

Column :Inertsil ODS-3 4.6 x 250 mm

Equipment: SS-CM-0310

<Chromatogram>

mV

min

0

5

10

15

20

25

0

250

500

750

1000

1250

Detector A Channel 1 220nm

1

1 / 13.858

2 / 14.383

3 / 14.725

<Peak Table>

Detector A Channel 1 220nm

| Peak# | Ret. Time | Area | Height | Area% |
| --- | --- | --- | --- | --- |
| 1 | 13.858 | 71666 | 5293 | 0.970 |
| 2 | 14.383 | 7286937 | 1208817 | 98.667 |
| 3 | 14.725 | 26746 | 4021 | 0.362 |
| Total |  | 7385349 | 1218130 | 100.000 |

##
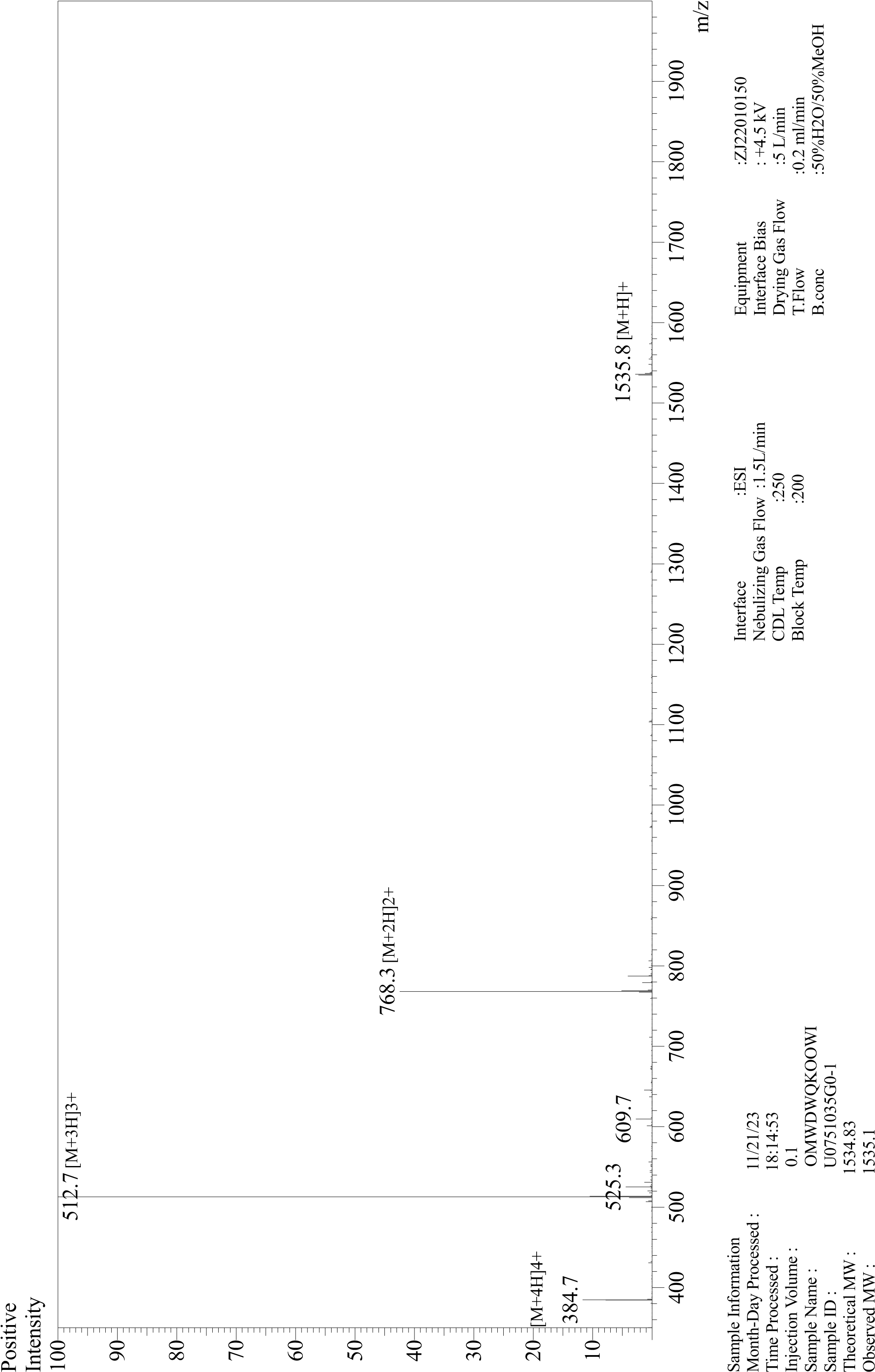
OMWDWQKOOWI, Mass Spectrometry

Mass Spectrum

## OMWDWQKOKWI, HPLC

Sample Name :OMWDWQKOKWI

Sample ID :U1186JGJG0-1

Time Processed :23:24:15

Month-Day-Year Processed :01/09/2024

Pump A : 0.065% trifluoroacetic in 100% water (v/v)

Pump B : 0.05% trifluoroacetic in 100% acetonitrile (v/v)

Total Flow:1 ml/min

Wavelength:220 nm

<<LC Time Program>>

| Time | Module | Command | Value |
| --- | --- | --- | --- |
| 0.01 | Pumps | Solvent B Conc. | 5 |
| 25.00 | Pumps | Solvent B Conc. | 65 |
| 25.01 | Pumps | Solvent B Conc. | 95 |
| 27.00 | Pumps | Solvent B Conc. | 95 |
| 27.01 | Pumps | Solvent B Conc. | 5 |
| 33.00 | Pumps | Solvent B Conc. | 5 |
| 33.01 | Controller | Stop |  |

<<Column Performance>>

<Detector A>

Column :Inertsil ODS-SP 4.6 x 250 mm

Equipment: ZJ19010015

<Chromatogram>

mV

min

0

5

10

15

20

25

0

500

1000

1500

Detector A Channel 1 220nm

1

1 / 7.233

2 / 9.658

3 / 10.042

4 / 10.175

5 / 10.617

6 / 10.967

7 / 11.192

8 / 11.400

<Peak Table>

Detector A Channel 1 220nm

| Peak# | Ret. Time | Area | Height | Area% |
| --- | --- | --- | --- | --- |
| 1 | 7.233 | 44961 | 5565 | 0.225 |
| 2 | 9.658 | 84044 | 10486 | 0.421 |
| 3 | 10.042 | 89510 | 9654 | 0.449 |
| 4 | 10.175 | 95660 | 10331 | 0.480 |
| 5 | 10.617 | 19284584 | 1760543 | 96.666 |
| 6 | 10.967 | 191535 | 16324 | 0.960 |
| 7 | 11.192 | 95309 | 10758 | 0.478 |
| 8 | 11.400 | 64108 | 4479 | 0.321 |
| Total |  | 19949713 | 1828140 | 100.000 |

##
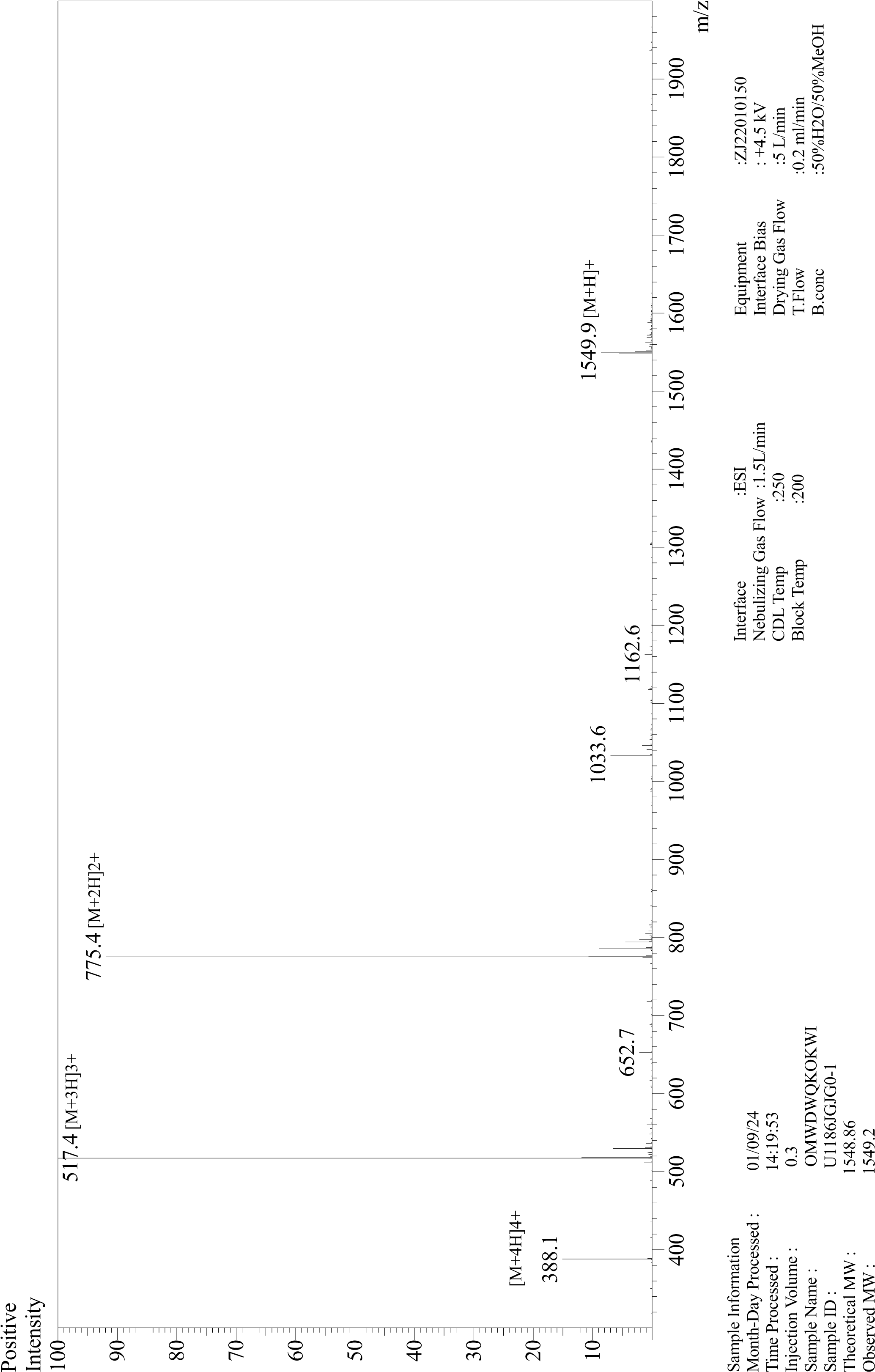
OMWDWQKOKWI, Mass Spectrometry

Mass Spectrum

# Supporting References

[1] T. R. Eykyn, G. S. Payne, M. O. Leach, *Phys. Med. Biol.* **2005**, *50*, N371–N376.

[2] X. Xu, J.-S. Lee, A. Jerschow, *Angew. Chem. Int. Ed.* **2013**, *52*, 8281–8284.

[3] M. Kim, J. Gillen, Bennett. A. Landman, J. Zhou, P. C. M. van Zijl, *Magn Reson Med* **2009**, *61*, 1441–1450.

[4] M. Gram, M. Seethaler, D. Gensler, J. Oberberger, P. M. Jakob, P. Nordbeck, *Magnetic Resonance in Medicine* **2021**, *85*, 2771–2780.

[5] L. Zhang, Y. Zhao, Y. Chen, C. Bie, Y. Liang, X. He, X. Song, *Quant Imaging Med Surg* **2019**, *9*, 1714–1730.

[6] M. T. McMahon, A. A. Gilad, J. Zhou, P. Z. Sun, J. W. M. Bulte, P. C. M. van Zijl, *Magnetic Resonance in Medicine* **2006**, *55*, 836–847.

[7] M. Zaiss, G. Angelovski, E. Demetriou, M. T. McMahon, X. Golay, K. Scheffler, *Magnetic Resonance in Medicine* **2018**, *79*, 1708–1721.

[8] J. Rey, S. Murail, S. de Vries, P. Derreumaux, P. Tuffery, *Nucleic Acids Research* **2023**, *51*, W432–W437.

[9] P. Tufféry, P. Derreumaux, *Front Bioinform* **2023**, *3*, 1113928.

[10] V. Binette, N. Mousseau, P. Tuffery, *J Chem Theory Comput* **2022**, *18*, 2720–2736.

[11] A. Lamiable, P. Thévenet, J. Rey, M. Vavrusa, P. Derreumaux, P. Tufféry, *Nucleic Acids Res* **2016**, *44*, W449–W454.

[12] O. Perlman, H. Ito, A. A. Gilad, M. T. McMahon, E. A. Chiocca, H. Nakashima, C. T. Farrar, *Sci Rep* **2020**, *10*, 20664.

[13] H. Nakashima, T. Nguyen, K. Kasai, C. Passaro, H. Ito, W. F. Goins, I. Shaikh, R. Erdelyi, R. Nishihara, I. Nakano, D. A. Reardon, A. C. Anderson, V. Kuchroo, E. A. Chiocca, *Clin Cancer Res* **2018**, *24*, 2574–2584.

[14] E. Liepinsh, G. Otting, K. Wüthrich, *J Biomol NMR* **1992**, *2*, 447–465.

[15] V. Khlebnikov, W. J. M. van der Kemp, H. Hoogduin, D. W. J. Klomp, J. J. Prompers, *Sci Rep* **2019**, *9*, 1089.

[16] M. Zaiss, P. Bachert, *Phys. Med. Biol.* **2013**, *58*, R221–R269.
